# Redefining *q*: quaternary ammonium cross sectional area (XSA) as a general descriptor for transport-limiting PTC rate approximations†
†Electronic supplementary information (ESI) available. See DOI: 10.1039/c5sc00071h



**DOI:** 10.1039/c5sc00071h

**Published:** 2015-02-12

**Authors:** S. E. Denmark, J. J. Henle

**Affiliations:** a Roger Adams Laboratory , Department of Chemistry , University of Illinois , Urbana , Illinois 61801 , USA . Email: sdenmark@illinois.edu

## Abstract

The relationships of several key molecular descriptors with the rate of the O'Donnell alkylation under phase transfer catalysis (PTC) have been investigated. The most common parameter used to predict PTC rates, the ammonium ion accessibility, ***q***, is defined in such a way that limits its use to straight-chain tetraalkylammonium catalysts. To find a general descriptor of rate, eight linear, symmetrical tetraalkylammonium cations were examined to determine if a model containing broadly applicable descriptors could be found. The catalytic activity of these salts was determined under PTC conditions (operating under an interfacial, transport-rate limiting mechanism) and was compared with these molecular descriptors. Models could be generated from the ammonium ion accessibility parameter ***q*** and the amphiphilic cross sectional area descriptor (XSA), and each gave a correlative model predicting the rate of alkylation. However, a similar model cannot be generated from a descriptor that is a direct measure of ammonium ion accessibility, the solvent accessible ammonium surface area (NC4_SA). These models lead to the conclusion that ***q*** must approximate catalyst properties other than ammonium ion accessibility. Additionally, the relationship between XSA and rate demonstrates that XSA approximates the complex behavior of ammonium ions at the interfacial region of a biphasic system, allowing for its use as a general descriptor for transport-limiting PTC rate approximations.

## Abstract 2

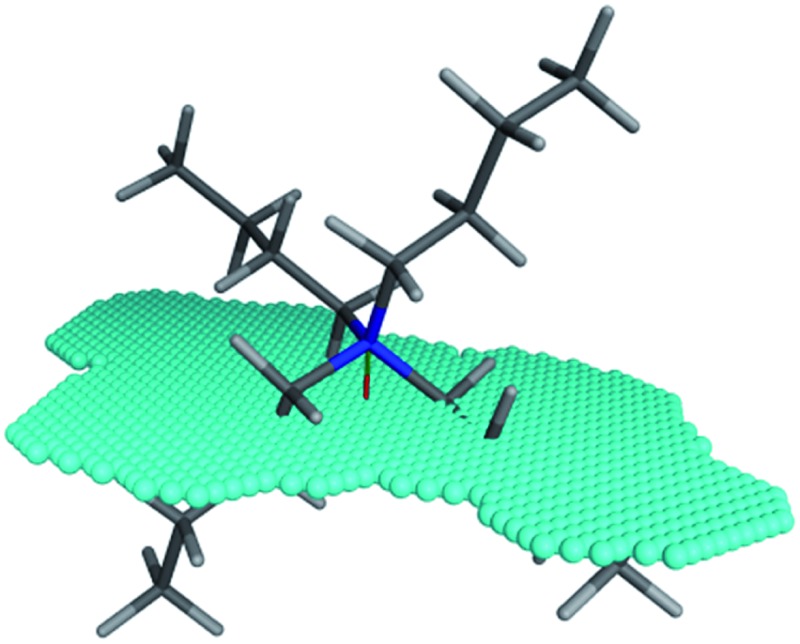
Several descriptors were studied in transport-rate limiting PTC, with amphiphilic cross-sectional area (XSA) identified as a single-descriptor model of rate.

## Introduction

Phase-transfer catalysis (PTC) is an extremely useful method for performing nearly any type of reaction involving an ionic starting material or intermediate with a neutral partner.^1^ In PTC, two immiscible phases separate reactive reagents. Reaction and catalysis is enabled by a phase-transfer reagent; typically a quaternary ammonium or phosphonium salt that facilitates the transfer of a reactive agent between the two immiscible phases. Several characteristics of PTC are attractive for industrial applications including ease of scalability, operational simplicity, and ease of separation of products from byproducts. A useful aspect of PTC is the ability to catalyze a wide variety of reaction types, ranging from redox processes to C–C bond forming reactions.^2^ An important type of C–C bond forming reaction is the alkylation of carbanions formed under PTC with inorganic hydroxide bases, termed “hydroxide-initiated PTC”.^3^ Employing hydroxide bases to generate anions that are typically formed using strong bases (LDA, NaH, *etc.*) highlights the advantages in operation and scalability using PTC. Hydroxide-initiated PTC has been highly successful in the catalytic, enantioselective alkylation of enolates.^4^

**Fig. 1:**
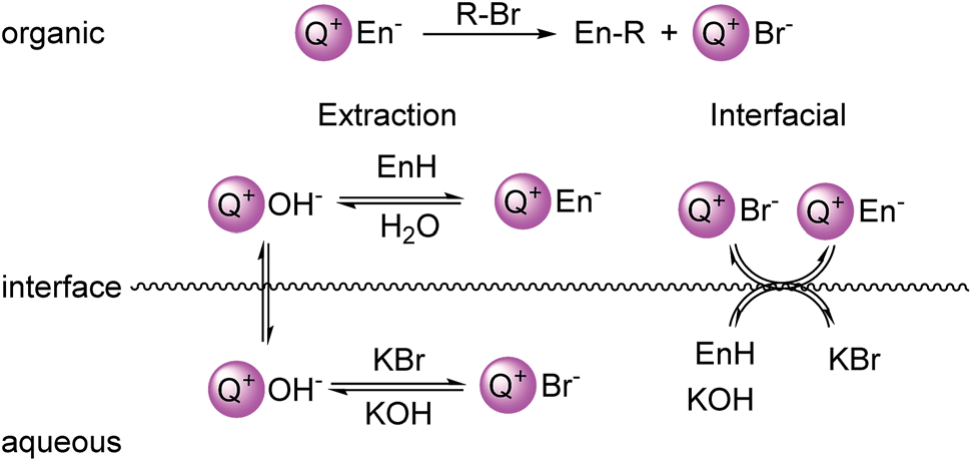
Left: depiction of the Stark's extraction mechanism for hydroxide-initiated PTC. Right: depiction of Makosza's interfacial mechanism for hydroxide-initiated PTC. En = enolate.

The versatility of PTC of reactions involving ionic intermediates places it at the forefront of general utility within organic synthesis. However, a fundamental understanding of the structural features that lead to catalytic activity and selectivity is still lacking. This deficiency arises from the inherent complexity of the roles of catalyst, base, and substrate in a biphasic mixture. For PTC to be successful, both physical (transport) and chemical (reactivity) aspects must be considered. In hydroxide-initiated PTC, two limiting mechanisms have been proffered to explain the activity of the catalyst: the extraction mechanism proposed by Starks^5^ and the interfacial mechanism proposed by Makosza^6^ (Fig. 1). The extraction mechanism postulates that quaternary ammonium species (**Q**^**+**^) acts to transfer hydroxide (**OH**^**–**^) to the organic phase to generate the ammonium enolate (**Q**^**+**^**En**^**–**^) (Fig. 1, left). In the interfacial mechanism, the enolate is generated at the interfacial region between the organic and aqueous phases by reaction with hydroxide (Fig. 1, right). The quaternary ammonium salt then effects ion exchange and transfers the enolate from the interfacial region (desorption) into the organic phase, where alkylation can take place. Numerous kinetic studies have provided evidence for both of these mechanisms, depending on reaction conditions.^7^ For practical purposes, a general guideline is that PTC reactions of carbon acids with a p*K*_a_ range 18 < *x* < 25 undergo reaction in the interfacial mode.

Despite extensive kinetic investigation, a predictor of catalyst activity in hydroxide-initiated PTC remains elusive as no general design parameters that lead to high catalytic activity have been identified. For PT-catalysts, structure–activity relationships (SAR) have been identified for straight-chain tetraalkylammonium (R_4_N^+^) salts that promote reaction of small hydrophilic nucleophiles. However, such studies involving hydroxide-initiated PTC are primitive by comparison.^1*e*^ The most commonly invoked structural features of R_4_N^+^ salts in SAR studies is the ammonium ion accessibility parameter ***q***.^8^ The parameter ***q*** is a structural descriptor of straight-chain R_4_N^+^ species originally introduced by Rabinovitz and Halpern in a series of studies that aimed to define the limits of the spectrum between the two aforementioned mechanisms.^9^ The parameter ***q*** is defined as the sum of the reciprocal of the number of carbons in the R_4_N^+^ chains (eqn (1), where *C*_*n*_ is the number of carbon atoms in chain *n*). After surveying many hydroxide-initiated PTC reactions, it was observed that most active catalysts had ***q***-values between 1.5 and 2.0.^10^ Generally, a R_4_N^+^ ion is considered accessible if ***q*** > 1.1
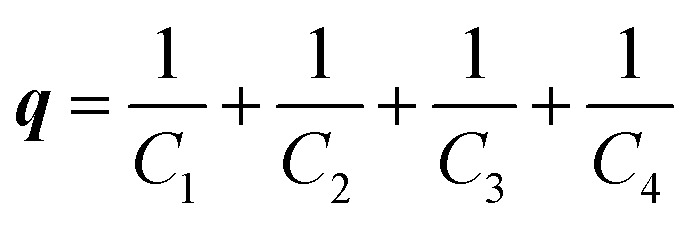



**Fig. 2:**
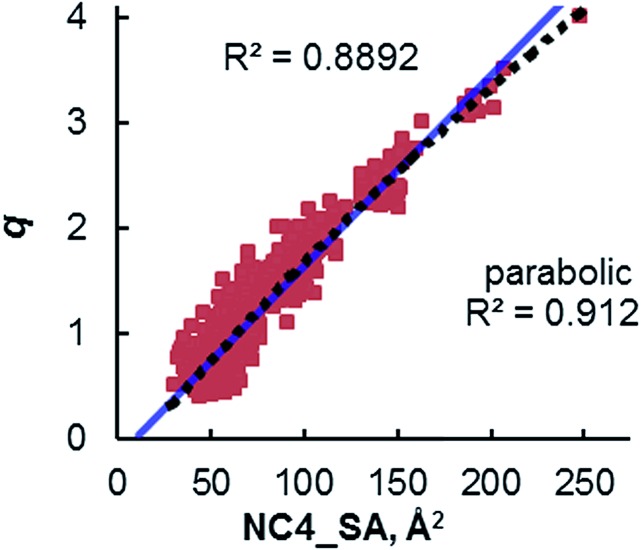
Relationship of the NC4_SA values of all straight-chain alkyl R_4_N^+^ catalysts containing 4–40 carbon atoms and ***q***. Adapted with permission from S. E. Denmark, N. D. Gould, L. M. Wolf, *J. Org. Chem.*, 2011, **76**, 4337–4357.

Although ***q*** may exist as a general descriptor for straight-chain R_4_N^+^ ion activity in hydroxide initiated PTC, the definition of the parameter prevents its use in the case of more complex quaternary ammonium species. As the logic behind the derivation of ***q*** was never clarified, extension of the parameter is difficult. To address this problem, previous work in these laboratories introduced a more general catalyst structure descriptor to correlate with rate data.^11^ This type of descriptor is of paramount importance in employing asymmetric PTC (APTC) reactions that require chiral, branched, and substituted quaternary ammonium species. The ability to select catalysts that can out-compete a background reaction is fundamental to chiral catalyst design. Although many descriptors were evaluated as potential replacements for ***q***, it was found that ***q*** correlates well with the solvent accessible ammonium ion surface area descriptor (NC4_SA, Fig. 2). The NC4_SA descriptor calculates the solvent accessible surface area (ASA) of the atoms that comprise the ammonium ion (nitrogen and neighboring carbon atoms), a direct measure of ammonium ion accessibility.^12^

**Fig. 3:**
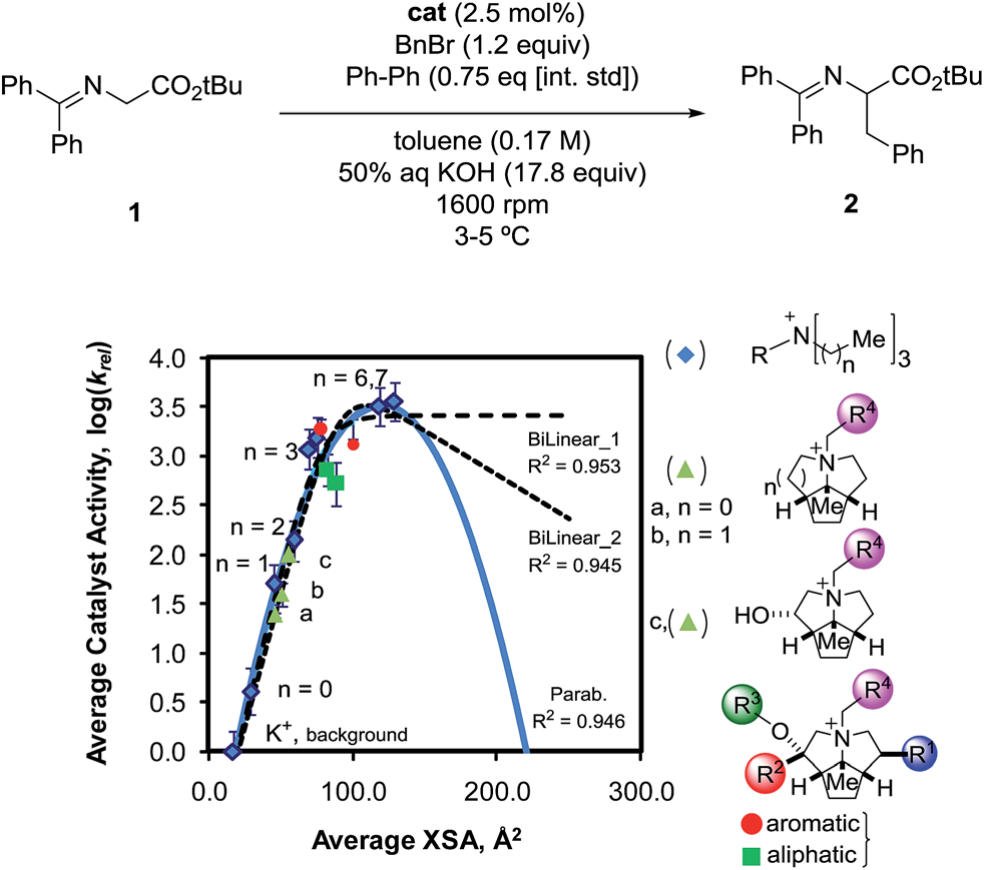
Observed correlation between XSA descriptor and catalyst activity in the O'Donnell alkylation. Adapted with permission from S. E. Denmark, N. D. Gould, L. M. Wolf, *J. Org. Chem.*, 2011, **76**, 4337–4357.

Once the correlation was established, a kinetic study was performed to investigate the relationship of NC4_SA and other molecular descriptors with rate. This previous work^11^ used the O'Donnell benzylation of glycine Schiff base **1** as a model reaction (Fig. 3) to study using these descriptors.^13^ Catalytic activity data was taken under conditions such that the reaction exhibited stir rate dependence with a highly reactive nucleophile ensuring that the reaction is transport-rate limiting (*i.e.* alkylation is *not* the rate-limiting step).^11c–e^ In the original report, it was shown that NC4_SA had a moderate correlation with the rate of the O'Donnell alkylation in conjunction with other molecular descriptors.^11^ However, it was found in the original study that the amphiphilic cross-sectional area^14^ descriptor (XSA) acted as a potential single descriptor for rate (Fig. 3) exceeding the correlation observed in rate models containing NC4_SA.

**Fig. 4:**
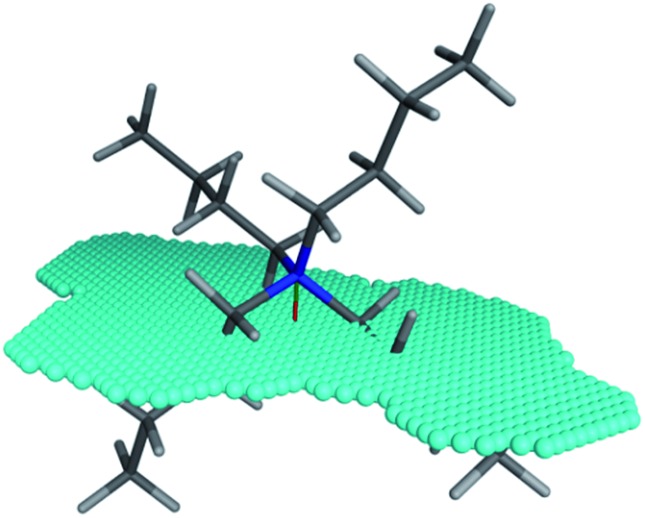
Amphiphilic cross sectional area (XSA) of the tetrabutylammonium cation. The amphiphilic axis is shown as the red-green line, and the cross section plane is denoted by the blue spheres.

The XSA descriptor first determines the amphiphilic axis (the axis between the center of mass of hydrophobic and polar atoms, respectively), then a plane is defined at the center of this axis, lying orthogonal to the axis. The area of this plane circumscribing the van der Waals surface of the molecule is the XSA value in Å^2^ (Fig. 4). The XSA descriptor models behavior of amphiphilic molecules at the highly anisotropic interface between a polar and nonpolar environment, and is thus ideally suited to model catalysts operating in the interfacial PTC mechanism.^15^

A linear increase of rate with increasing XSA was observed until 75 Å^2^ was reached, and which point the reaction rate leveled off, with the larger R_4_N^+^ salts having a maximum area of 131 Å^2^. The original work concluded that over the observed range, XSA is proportional to the rate at which the **Q**^**+**^**En**^**–**^ species can undergo the desorption process from the interfacial region. It is proposed that the levelling of rate results from the rate of desorption approaching the diffusion limit. From this data, three nonlinear correlative models were found, shown in Fig. 3.

Depending on the model considered, different interpretations of what XSA physically represents are possible. If the constant rate trend was found to continue (Fig. 3, Bilinear_1), it would indicate that XSA is solely modeling desorption from the interface. If the rate began decreasing as XSA increases (fitting models similar to Bilinear_2 and Parab, Fig. 3), this would indicate that XSA models adsorption to and desorption from the interface. The goal of the study herein was to determine the kinetic behavior of PTC catalysts in a higher (>130 Å^2^) XSA regime in the O'Donnell benzylation reaction and determine to which model, if any, the kinetic data conforms. Additionally, other descriptors such as ***q*** and NC4_SA were also investigated for correlative relationships with PTC rates.

## Results and discussion

**Fig. 5:**
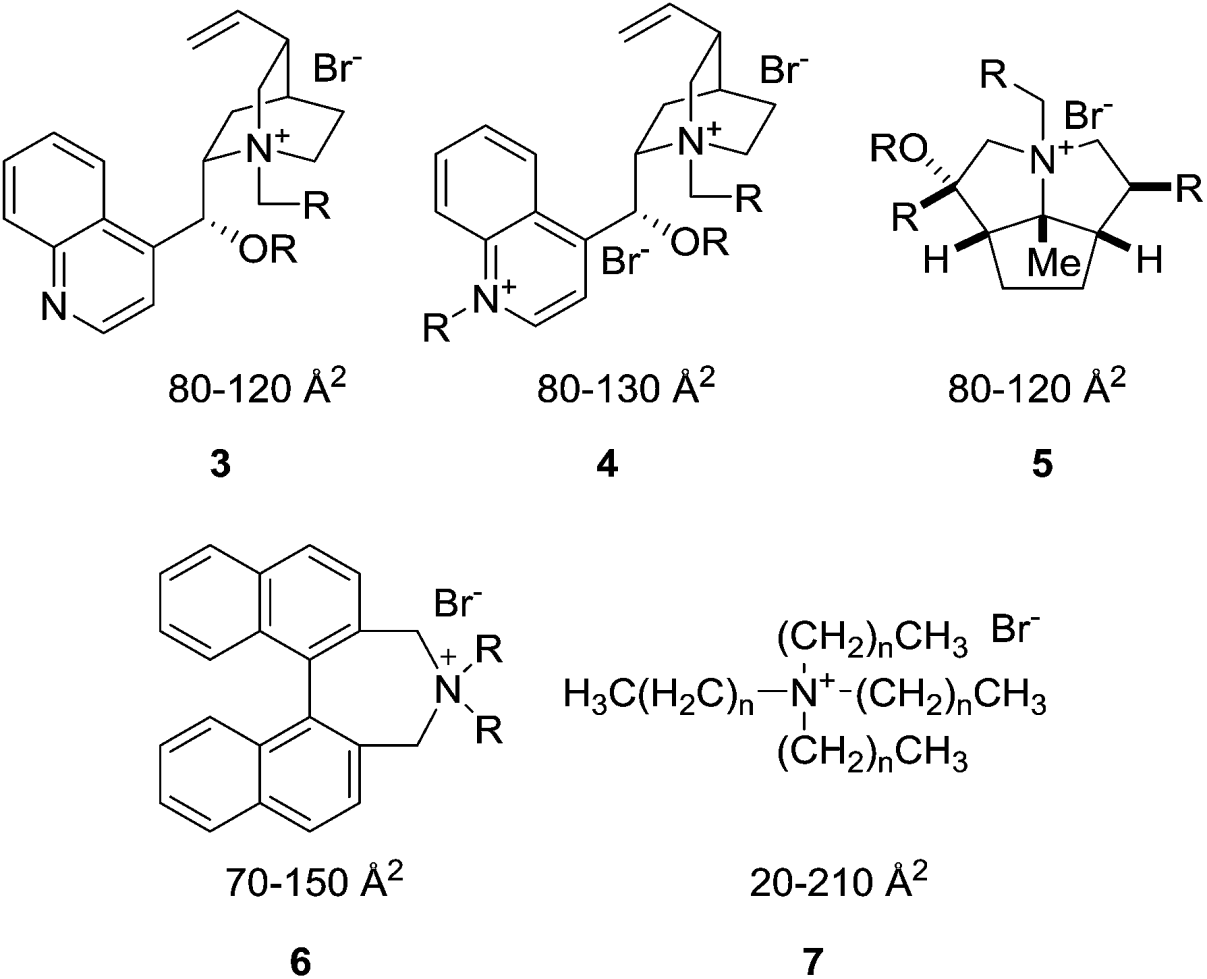
The tetraalkylammonium salts from a variety of scaffold libraries used for *in silico* screening of various descriptors. The values given are the general ranges of XSA values for each scaffold. These values are calculated using the method described in the ESI.†

To further elucidate the physical interpretation of the XSA descriptor with respect to transport-limiting PTC, catalysts possessing larger XSA's would be evaluated for PTC activity. Quaternary ammonium catalysts were identified through a selective *in silico* quaternary ammonium ion screen. The library consisted of candidates derived from the common unsymmetrical, complex R_4_N^+^ cores (*Cinchona* alkaloids (**3**, **4**), cyclopentapyrrolizidines (**5**),^11^ Maruoka catalysts (**6**),^16^ *etc.*) as well as commercially available straight-chain R_4_N^+^ salts (**7**) (Fig. 5).

None of the complex, polycyclic scaffolds yielded structures with calculated XSA's greater than 150 Å^2^. However, several, large XSA salts were found in the straight-chain R_4_N^+^ library. In view of their commercial availability, tetradecylammonium bromide (TDAB), tetradodecylammonium bromide (TDoDAB), and tetrakis(hexadecyl)ammonium bromide (THexDAB) were chosen as the large XSA catalyst representatives (Table 1, entries 6–8). In addition, it was deemed prudent to reassess the activity of the straight-chain R_4_N^+^ catalysts previously reported to ensure that accurate correlations were obtained. The calculated average XSA values are reported in Table 1.^17^

**Table 1:** Amphiphilic cross sectional area (XSA) and solvent accessible ammonium ion surface area (NC4_SA) values for the straight-chain R_4_N^+^ salts

Entry	Catalyst (R_4_N^+^)	Catalyst	XSA^*a*^ ^,^^*b*^ (Å^2^)	NC4_SA^*a*^ ^,^^*b*^ (Å^2^)
1	(CH_3_)_4_N^+^	TMAB	29.00	247.24
2	(C_2_H_5_)_4_N^+^	TEAB	41.81	94.88
3	(C_4_H_9_)_4_N^+^	TBAB	72.22	52.23
4	(C_6_H_13_)_4_N^+^	THexAB	101.17	48.22
5	(C_7_H_15_)_4_N^+^	THeptAB	114.14	48.26
6	(C_8_H_17_)_4_N^+^	TOAB	128.83	47.68
7	(C_10_H_21_)_4_N^+^	TDAB	152.03	44.64
8	(C_12_H_25_)_4_N^+^	TDoDAB	174.34	39.10
9	(C_16_H_33_)_4_N^+^	THDAB	205.27	23.08

^*a*^Calculated using MOE 2013.08 using MMFF94x.

^*b*^Values are averages calculated from a conformer library containing conformers within 20 kcal mol^–1^ from equilibrium conformer.

**Fig. 6:**
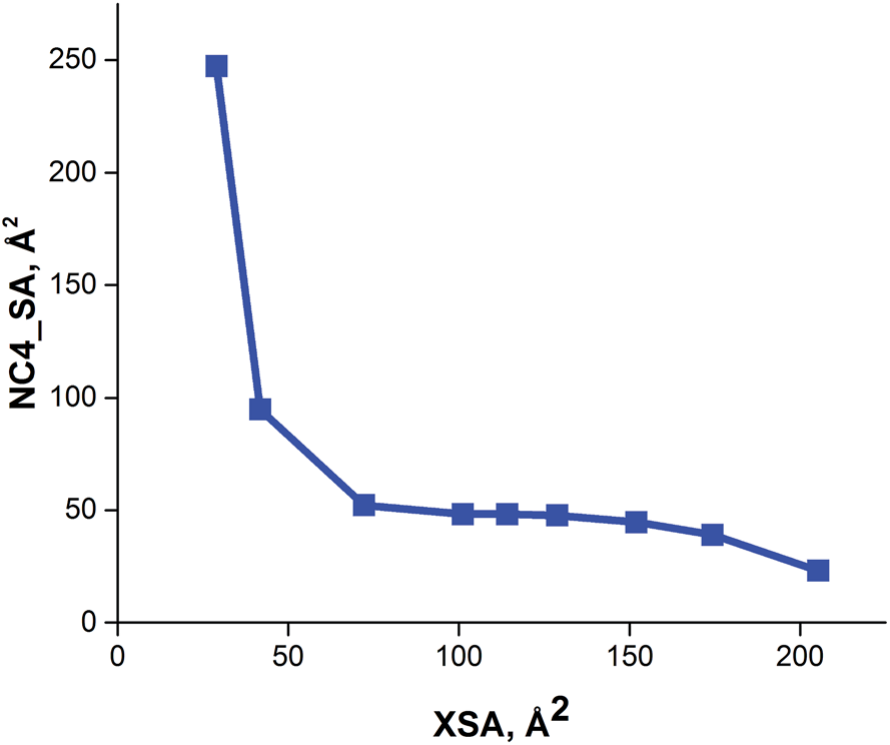
Relationship between cross sectional area (XSA) and solvent accessible ammonium ion surface area (NC4_SA) descriptors.

The solvent accessible ammonium ion surface area (NC4_SA) was also calculated for these large R_4_N^+^ catalysts. The results of these calculations show that the ammonium surface area remains invariant for larger values of XSA (Table 1). Thus, as XSA increases with increasing chain length in the R_4_N^+^ species, a decrease in NC4_SA is observed, followed by a relatively constant region once XSA ∼ 75 Å^2^ is reached (Fig. 6). This suggests that PT-catalysts with increasingly large XSA have similar ammonium ion accessibilities.

The selected straight-chain R_4_N^+^ salts were then employed as catalysts in the O'Donnell benzylation reaction. Each catalyst was tested in triplicate, with the resulting data having an average percent error of 3.19%.^18^ As in the previous study, the metric of catalytic activity was the half-life (*t*_1/2_) of the reaction at a constant stirring rate. The kinetic data obtained for these catalysts is shown in Table 2. Once again, the catalysts with the highest activity were THAB, THeptAB (heptyl) and TOAB (entries 3–5). After TOAB, an increase in the chain length of the R_4_N^+^ catalysts resulted in decreased activity (entries 6–8).

**Table 2:** Kinetic data for R_4_N^+^ PTC catalysts^*a*^

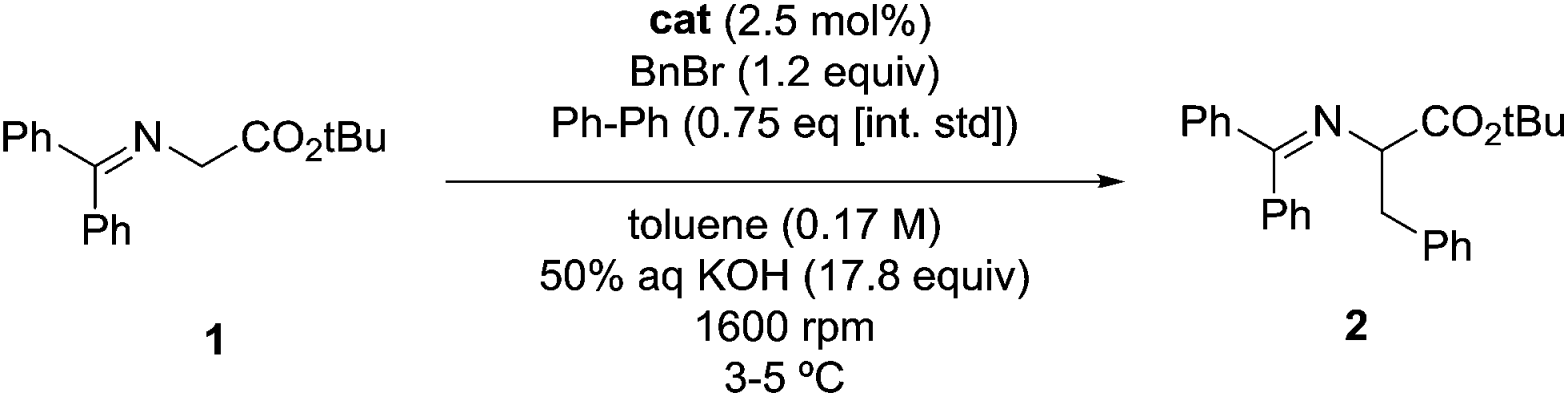				
Entry	Catalyst	Avg^*b*^ *t*_1/2_ (min)	log (*k*_rel_)	Error^*c*^
1	TMAB	2056.77	0.66	1.67%
2	TEAB	142.68	1.82	1.56%
3	TBAB	13.79	2.83	3.00%
4	THexAB	9.01	3.02	4.50%
4	THeptAB	9.50	3.00	7.53%
5	TOAB	12.09	2.89	5.75%
6	TDAB	81.80	2.06	2.27%
7	TDoDAB	413.12	1.36	0.67%
8	THexDAB	6302.73	0.17	1.74%
9	None	9411.2	0	1.67%
			Avg err	3.19%

^*a*^All reactions performed on a 0.34 mmol scale.

^*b*^Average of at least three runs.

^*c*^Error calculated as stddev(log(*t*_1/2_))/log(*t*_1/2_)_avg_ and reported as percentage.

**Fig. 7:**
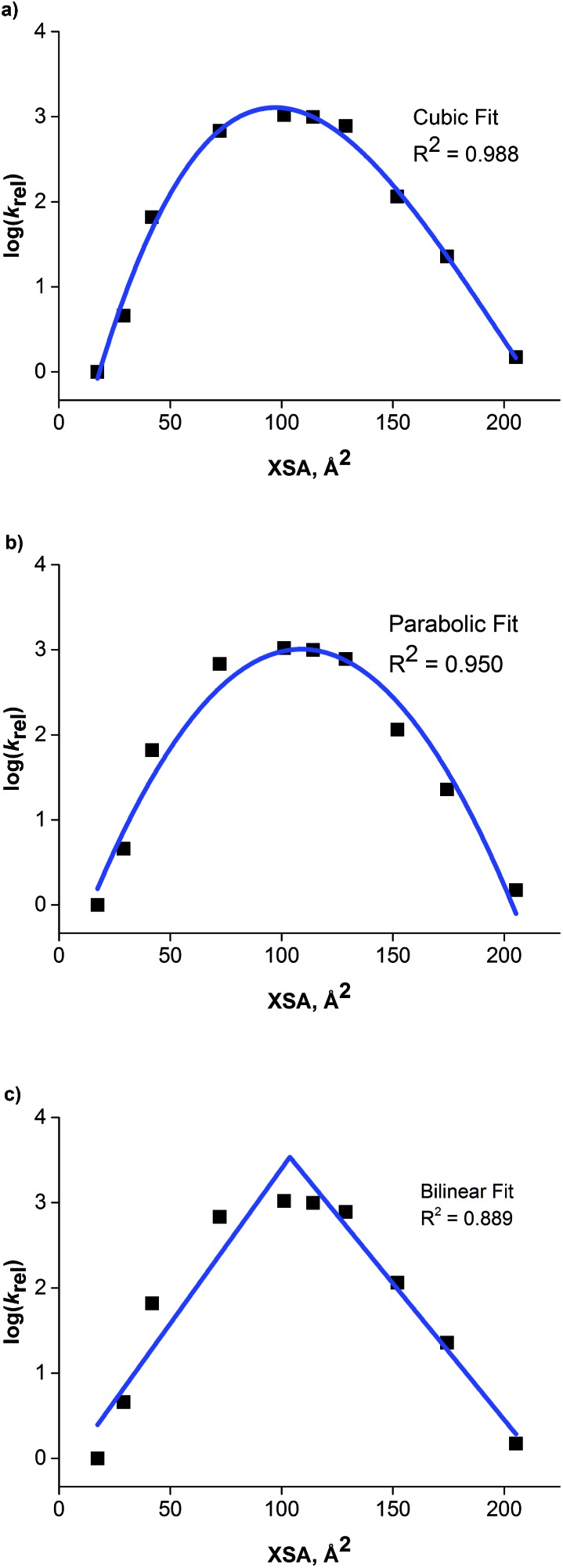
(a) Cubic model between XSA and log(*k*_rel_). (b) Parabolic model between XSA and log(*k*_rel_). (c) Bilinear model between XSA and log(*k*_rel_).

The relationship between the rate data and XSA was examined. Clearly, the rate does not remain constant (after reaching the maximum observed rate) with increasing XSA, but instead, a nonlinear relationship is observed is observed between rate and XSA (Fig. 7). The best^19^ model was observed with a third order polynomial (cubic function) with an *R*^2^ of 0.988 (Fig. 7a).^20^ A parabolic model was generated, giving a similarly high *R*^2^ of 0.950 (Fig. 7b). A bilinear model was investigated, but gave poorer observed correlation (*R*^2^ = 0.889) (Fig. 7c). While the parabolic model shows good correlation, the cubic model appears to more accurately describe the behavior of the most active catalysts as well as the least active.^19^ This is due to the change of rate with respect to XSA being non-symmetrical between the low XSA and high XSA catalyst regimes.

**Fig. 8:**
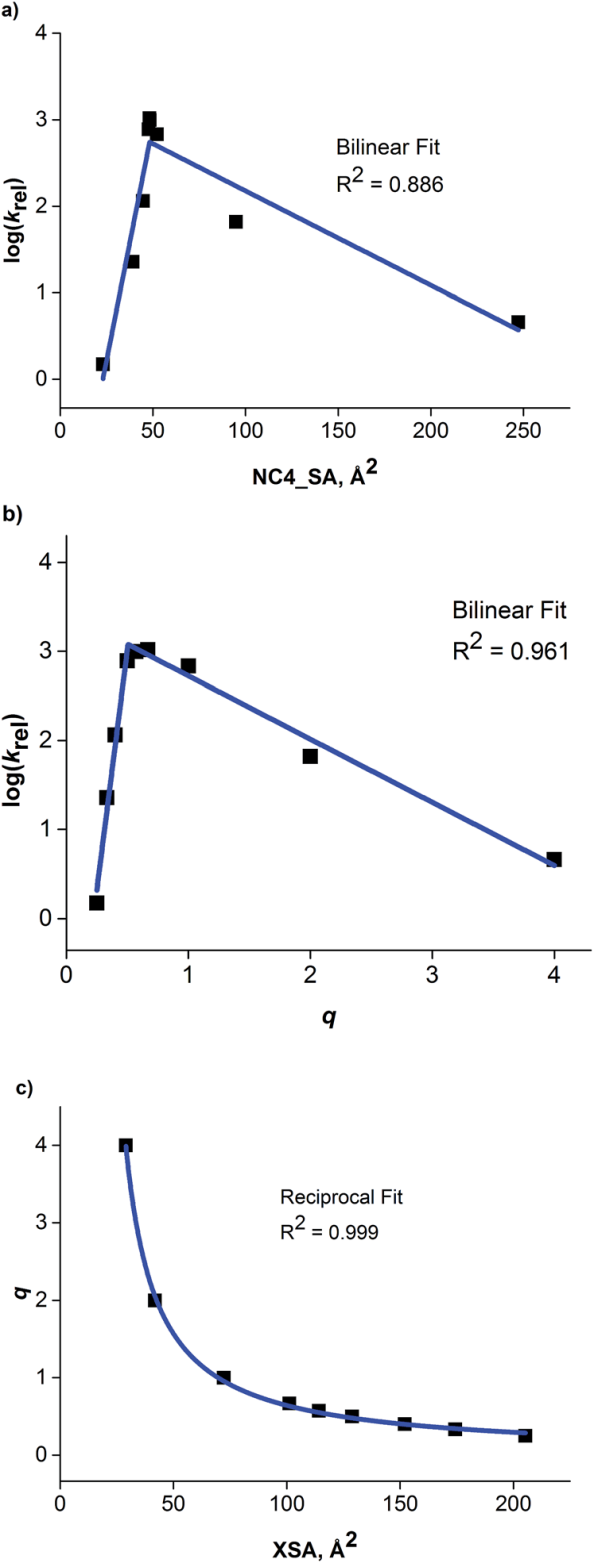
(a) Plot of NC4_SA against observed rate. (b) Bilinear relationship observed between ammonium ion accessibility (***q***) and observed rate. (c) Reciprocal relationship between XSA and ***q***.

The correlation between ammonium ion accessibility and rate was investigated using the solvent accessible ammonium ion surface area (NC4_SA) and ***q*** descriptors. Plotting the rate data with respect to the solvent accessible charged surface area (NC4_SA) showed a moderate bilinear correlation with an *R*^2^ of 0.886 (Fig. 8a). The lack of NC4_SA variation for the longer-chain R_4_N^+^ catalysts results in an insufficient model. Interestingly, a bilinear relationship between ***q*** and rate does exist, with a high degree of correlation (*R*^2^ of 0.961) (Fig. 8b). The fit is not as precise as that observed between XSA and log(*k*_rel_), but the correlation exists nonetheless.

The existence of a highly correlated bilinear relationship between ***q*** and rate is perplexing in view of the relatively poor correlation observed between NC4_SA and rate. The NC4_SA descriptor is an actual measure of ammonium ion accessibility and if ***q*** measured ammonium ion accessibility, it would be expected to give a moderately correlated model as well. Thus, the superior correlation of ***q*** and rate indicates that the ***q*** descriptor, while operationally simple in calculation, encompasses a wider range of properties than ammonium ion accessibility in the traditional sense.

The existence of the relationships between rate, XSA, and ***q*** suggested a correlation between XSA and ***q***. Indeed, an excellent reciprocal model (*R*^2^ = 0.999) between XSA and ***q*** was found (Fig. 8c). This correlation indicates that for the straight-chain R_4_N^+^ catalysts, ***q*** is an approximation of the inverse of XSA. The reciprocity arises due to catalysts with large XSA values generally containing long alkyl chains, giving a smaller value of ***q***.^21^ This leads to the conclusion that ***q*** is not simply a measure of ammonium ion accessibility, but instead a measure of molecular behavior at an anisotropic interface between polar and nonpolar solvation regions; a set of properties XSA was designed to represent as a descriptor.

**Fig. 9:**
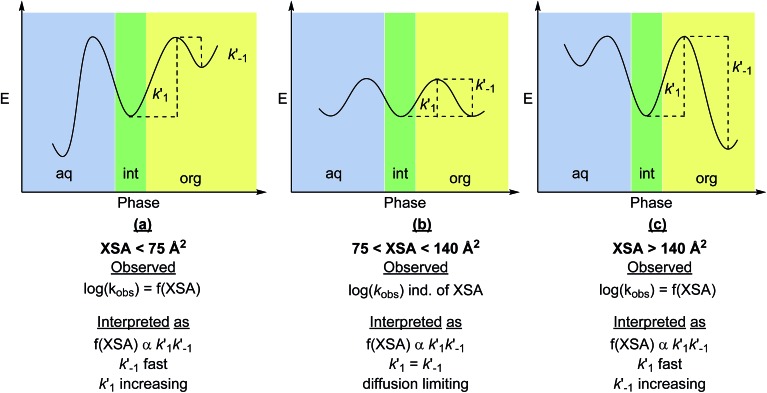
Physical interpretation of XSA with relationship to transport-rate limiting PTC mechanism: (a) desorption is the transport-rate limiting step for small R_4_N^+^ salts. (b) Both desorption and adsorption processes are diffusion rate limited (fast). (c) Adsorption becomes the transport-rate limiting step as the R_4_N^+^ become larger.^20^ The function *f*(XSA) is representative of the cubic model obtained between XSA and log(*k*_rel_). Note that the physical interpretation of positive and negative slope regimes is not dependent on the model used to predict the rate.

The observed correlations suggest that XSA describes the behavior of R_4_N^+^ ions, specifically the ability to adsorb and desorb from the interface. Envisioning the adsorption/desorption process as an equilibrium between the ion pair adsorbed at the interface and the ion pair residing in the organic phase leads to two transport-rate constants, *k*′1 (desorption) and *k*′–1 (adsorption) (Fig. 9). The observed rate (*k*_obs_) arises from a combination of *k*′1 and *k*′–1 The model obtained suggests that log(*k*_obs_) is correlated to XSA. The relationship between XSA and rate, which contains both positive and negative slopes, indicates a change in the transport-rate limiting step in the mechanism of the reaction. It should be noted that the non-linearity of the rate changes and the subsequent cubic model selection does not change the physical interpretation that follows, as this is based on the change in positive to negative slopes as XSA increases, and not on the rate of change of the slope.^22^

In the positive slope regime (XSA < 75 Å^2^), desorption from the interface (*k*′1 in Fig. 9a) is the expected slow step as observed in the original study. As the smaller R_4_N^+^ catalysts are hydrophilic (and less soluble in organic solvents), the energy barrier to achieve desorption from the interface is larger than the analogous process for adsorption to the interface, and thus the rate of desorption is the transport-rate limiting step. In the negative slope regime (XSA > 140 Å^2^), decreasing rate with increasing XSA suggests that adsorption to the interface (*k*′–1 in Fig. 9c) is the transport-rate limiting step. Since desorption should continue to be fast for the more lipophilic catalysts (*k*′1 is large), slow adsorption of the catalyst to the interface must be responsible for the decrease in rate; the energy barrier to achieve adsorption is likely lower for the smaller, more hydrophilic catalysts. The similar rates of catalysts at the inflection region (75 < XSA < 140 Å^2^) indicates that the rate of adsorption and desorption of the ion pairs are both fast, and likely diffusion limited (Fig. 9b). The trends in rate with respect to catalyst suggest that *k*_obs_ is proportional to *k*′1*k*′–1.

Because of the design of the XSA parameter as an estimation of molecular behavior under highly anisotropic conditions at an interface between polar and nonpolar environments and the observed correlation between XSA and rate, it is proposed that XSA represents the affinity of the catalyst at the interface (adsorption to interface) as well as affinity of the catalyst-enolate (**Q**^**+**^**En**^**–**^) ion pair for the organic phase (desorption from interface) (XSA correlates with *k*′1*k*′–1).^23^ The proper balance of these factors leads to high catalytic activity, which is observed in the 75 < XSA < 140 Å^2^ region; the highest rates are observed with catalysts of intermediate XSA, which likely exhibit diffusion rate limited transfer to and from the interface. Moreover, because of the correlation between ***q*** and XSA, it can be proposed that ***q*** is not a simple measure of the accessibility of the ammonium ion, but rather a reflection of the behavior of the ions at the interface. As shown, XSA appears to measure these same fundamental properties, but with better correlation with rate. Most importantly the XSA model can be applied to all types of ammonium ions. This feature means that XSA may be used to select and predict highly active catalysts. The previous report showed that XSA displayed the same correlation for rate with several cyclopentapyrrolizidine and *cinchona* type PTC catalysts.^11^ Unfortunately, the lack of larger XSA catalysts in these subclasses precludes the ability to explore further correlation in the high XSA regime.

## Conclusions

The correlation observed between XSA and log(*k*_rel_) serves to validate the prediction made in the initial report from these laboratories. By investigating quaternary ammonium salts with larger cross-sectional areas, a cubic (non-linear) relationship has been found between XSA and rate, indicating that XSA is sufficient as a single descriptor to predict transport-limiting PTC rates. Furthermore, the observed relationship with rate indicates that XSA captures the complicated behavior of the **Q**^**+**^ species at the interface in transport-limited PTC reactions of enolates. The observed relationship between ***q*** and both rate and XSA indicates that ***q*** models the same interfacial behavior instead of the physical accessibility of the ammonium ion. The observed correlations and derived models lead to the conclusion that XSA is an excellent surrogate for ***q*** in more complicated quaternary ammonium ion species. Further investigation will be required to show the utility of XSA to predict rate in other transport-limiting PTC reactions.

## Supplementary Material

Supplementary information
