# Supplementary material for: Redefining q: quaternary ammonium cross sectional area (XSA) as a general descriptor for transport-limiting PTC rate approximations
Source: Chem Sci. 2015 Feb 12;6(4):2211–false. doi: 10.1039/c5sc00071h (PMC5644375; doi:10.1039/c5sc00071h)
Supplement: Supplementary file 1 [file SC-006-C5SC00071H-s001.pdf]

Electronic Supplementary Material (ESI) for Chemical Science.
This journal is © The Royal Society of Chemistry 2015

Redefining *q*: Quaternary Ammonium Cross Sectional Area (XSA) as a General Descriptor for Transport-Limiting PTC Rate Approximations

Scott E. Denmark* and Jeremy J. Henle

Roger Adams Laboratory, University of Illinois, 600 S. Mathews Ave, Urbana, Illinois 61801

SUPPORTING INFORMATION

TABLE OF CONTENTS **PAGE**

General Experimental *S2*
General Reaction Procedure for Kinetic Reactions *S2*
Aliquot Procedure: *S3*
Tabulated Kinetic Data *S4*
Tetramethylammonium Bromide (TMAB) *S4*
Tetraethylammonium Bromide (TEAB) *S7*
Tetrabutylammonium Bromide (TBAB) *S10*
Tetrahexylammonium Bromide (THAB) *S15*
Tetraheptylammonium Bromide (THepAB) *S20*
Tetraoctylammonium Bromide (TOAB) *S23*
Tetradecylammonium Bromide (TDAB) *S26*
Tetradodecylammonium Bromide (TDoDAB) *S29*
Tetrakis(hexadecyl)ammonium bromide (THexDAB) *S32*
Background *S35*
Computational Methods and Data *S38*
Descriptors *S38*
Conformation Generation *S39*
XSA Descriptor Calculation *S40*
Ammonium SA Descriptor Calculation *S41*
Model Generation *S45*
References *S49*

General Experimental

Tetramethylammonium bromide (TMAB) was purchased from Aldrich, recrystallized from refluxing ethanol, dried under high vacuum and stored in an argon glove box. Tetraethylammonium bromide (TEAB) was purchased from Aldrich, powdered and dried under high vacuum and stored under argon prior to use. Tetrabutylammonium bromide (TBAB) was purchased from Aldrich and used as is. Tetrahexylammonium bromide (THAB) was purchased from Aldrich, washed with ether, dried under high vacuum for three days, and stored under argon. Tetraheptylammonium bromide (THepAB) was purchased from Across and used as is. Tetraoctylammonium bromide (TOAB), tetradodecylammonium bromide (TDoDAB), and tetrakis(hexadecyl)ammonium bromide (THexDAB) were purchased from Aldrich and used as is. Tetradecylammonium bromide (TDAB) was purchased from TCI and used as is. Benzyl bromide was purchased from Aldrich and purified by passing it through a plug of activity I basic alumina, and storing over 4Å mol sieves. The internal standard, biphenyl, was recrystallized from petroleum ether (40-60 ºC) prior to use.

Analytical high performance liquid chromatography (HPLC) was performed using an Agilent 1100 Series HPLC equipped with a variable wavelength detector (VWD). HPLC Method: 5 µL sample injections were made onto a Zorbax 3.5 µm column. Solvent flow rate maintained at 0.6 mL/min. Gradient elution used: 70:30□10:90, H_2O:MeCN, gradient over ten min. Hold for 5 min, then 70:30 H_2O:MeCN for 2 min, total run time 18 min.

Reactions were performed using a calibrated IKA color squid stir plate. Calibration was conducted with the help of the University of Illinois at Urbana-Champaign, School of Chemical Sciences Electronics Shop. Stir rate speeds were calibrated across the entire RPM range (0-2500 RPM) with the aid of a photo-tachometer, measuring the speed at which a stir bar was rotated.

General Reaction Procedure for Kinetic Reactions

All reaction vials and stir bars were acid washed, base washed, rinsed with deionized water and acetone, and oven dried for at least 12 h prior to use in kinetic reactions.

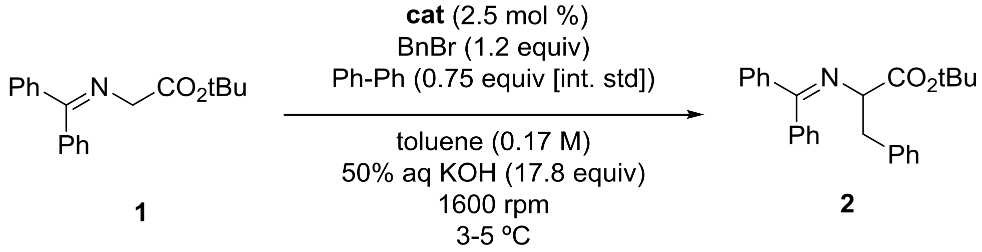

In a one-dram glass vial equipped with plastic cap, Teflon septum, and 1.5 cm x 0.5 cm football shaped stir bar was added *N*-(diphenylmethylene)glycine *tert*-butyl ester (100 mg, 0.34 mmol) and tetraalkylammonium bromide (0.0085 mmol, 0.025 equiv). To this was added 0.8 mL of benzyl bromide in toluene (69.5 mg, 0.41 mmol, 1.2 equiv, 87.1 mg/mL in toluene), followed by addition of 0.8 mL of biphenyl solution (40.7 mg, 0.264 mmol, 0.75 equiv, 50.83 mg/mL in toluene) as an internal standard. An additional 400 µL of toluene was added to the vial. The reaction solution was stirred (800 rpm) in a cold room maintained at 3-5 ºC for at least 1 h to achieve temperature equilibration. Following the equilibration period, the stir rate was increased to 1600 rpm and 660 uL of 50% aqueous KOH that had been pre-equilibrated to temperature was

added briskly. The timing of reaction began simultaneously with addition of base, which occurred over less than 5 s. Aliquots were then taken at appropriate time intervals to be analyzed by analytical HPLC.

Aliquot Procedure:
Two seconds prior to aliquot sampling, the stir plate was shut off to provide time for the layers to separate. At the time point, 5-7 µL of organic phase were removed using a 25 µL gas-tight syringe, and the stirring was restarted. The aliquot was quenched into a vial containing approximately 1-1.2 mL acetonitrile containing approximately 5 µL acetic acid. Prior to analysis, the aliquot solution is passed through a silica plug 5 mm by 15 mm.

Tabulated Kinetic Data
Tetramethylammonium Bromide (TMAB)

Run 1

Time (min)	Standard, µmol	Standard, area	Product, area	Product µmol	Product %
0	263.69	1.00	0.00	0	0.00
60	263.69	2601.14	134.49	15.43	4.59
120	263.69	3912.66	205.74	15.70	4.67
1221	263.69	3774.49	1378.77	109.04	32.42
1440	263.69	417.56	185.35	132.49	39.39
1690	263.69	6130.26	3052.99	148.65	44.20
2628	263.69	4138.83	2670.74	192.61	57.27

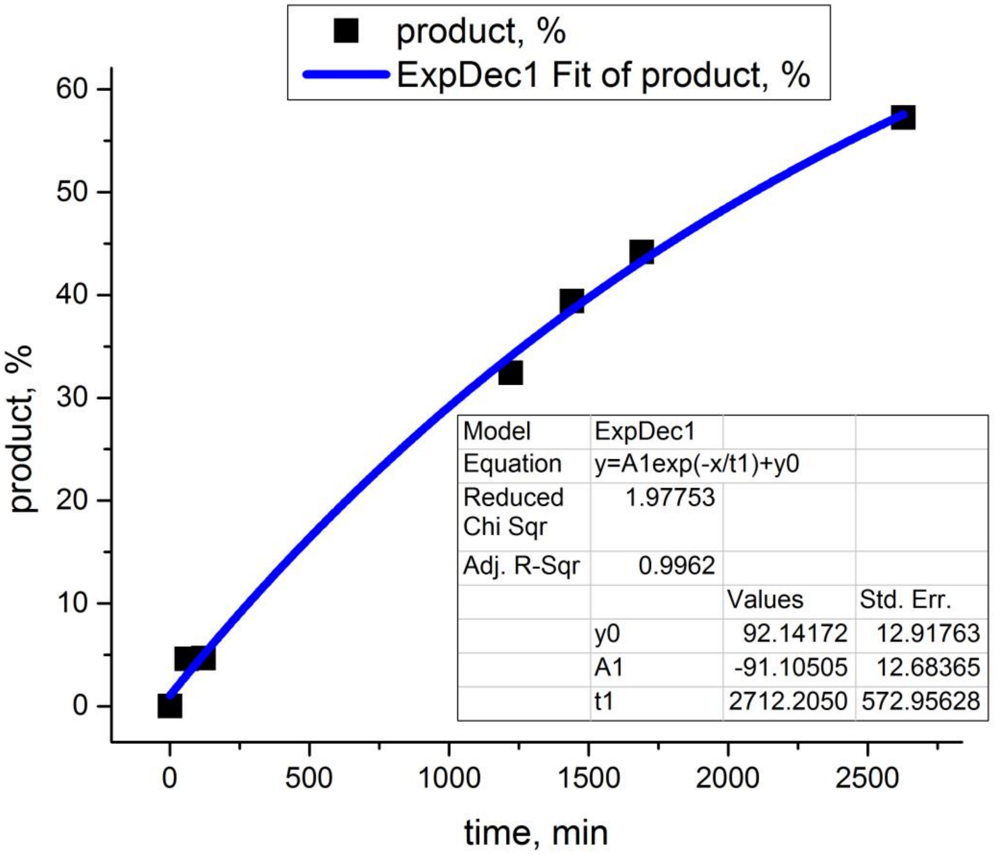

Interpolated $t_{1/2}$: 2091.04

Run 2

Time (min)	Standard, µmol	Standard, area	Product, area	Product µmol	Product %
0	263.69	1.00	0.00	0.00	0.00
60	263.69	5766.24	283.35	14.67	4.33
120	263.69	4932.06	242.34	14.67	4.33
1221	263.69	5381.84	1914.41	106.18	31.33
1440	263.69	3296.09	1382.10	125.16	36.93
1690	263.69	4663.19	2129.75	136.33	40.22
2628	263.69	6201.97	3831.96	184.43	54.41

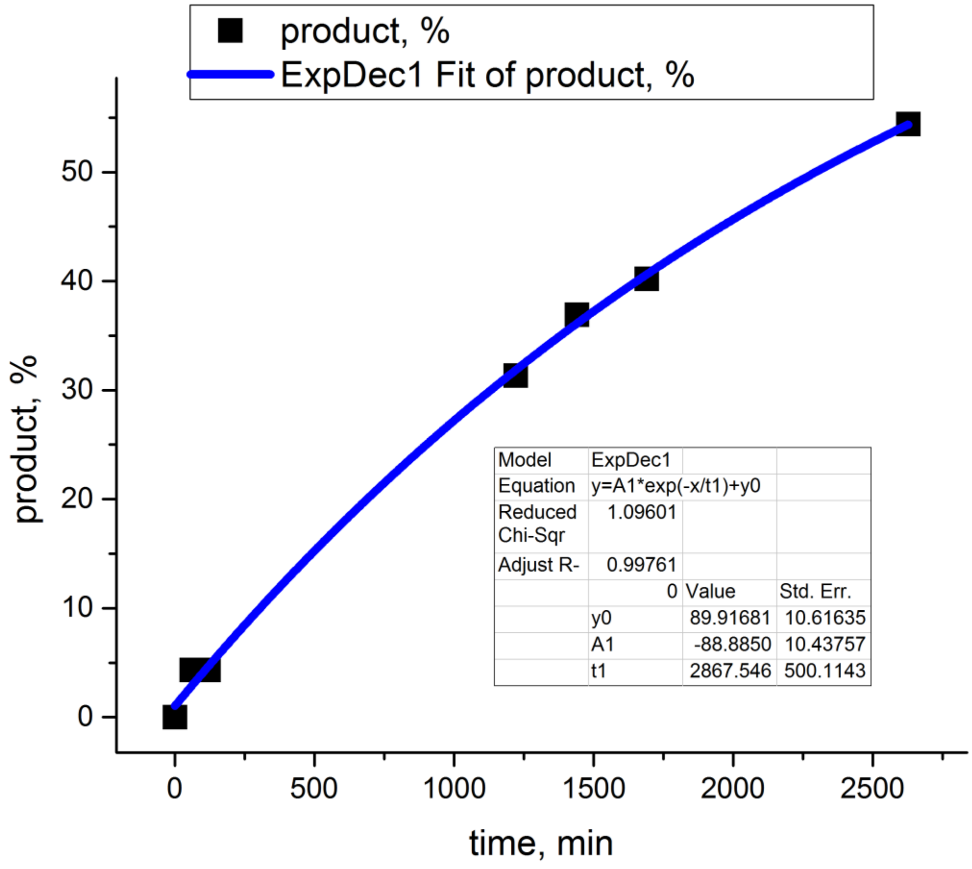

Interpolated $t_{1/2}$: 2295.60

Run 3

Time (min)	Standard, µmol	Standard, area	Product, area	Product µmol	Product %
0	263.69	1.00	0.00	0.00	0.00
60	263.69	5087.91	240.84	14.13	4.17
120	263.69	4617.23	283.35	18.32	5.40
1221	263.69	4404.30	1883.70	127.66	37.63
1440	263.69	4177.04	2075.09	148.29	43.71
1690	263.69	5644.26	3116.28	164.80	48.58
2628	263.69	4491.88	3199.86	212.64	62.68

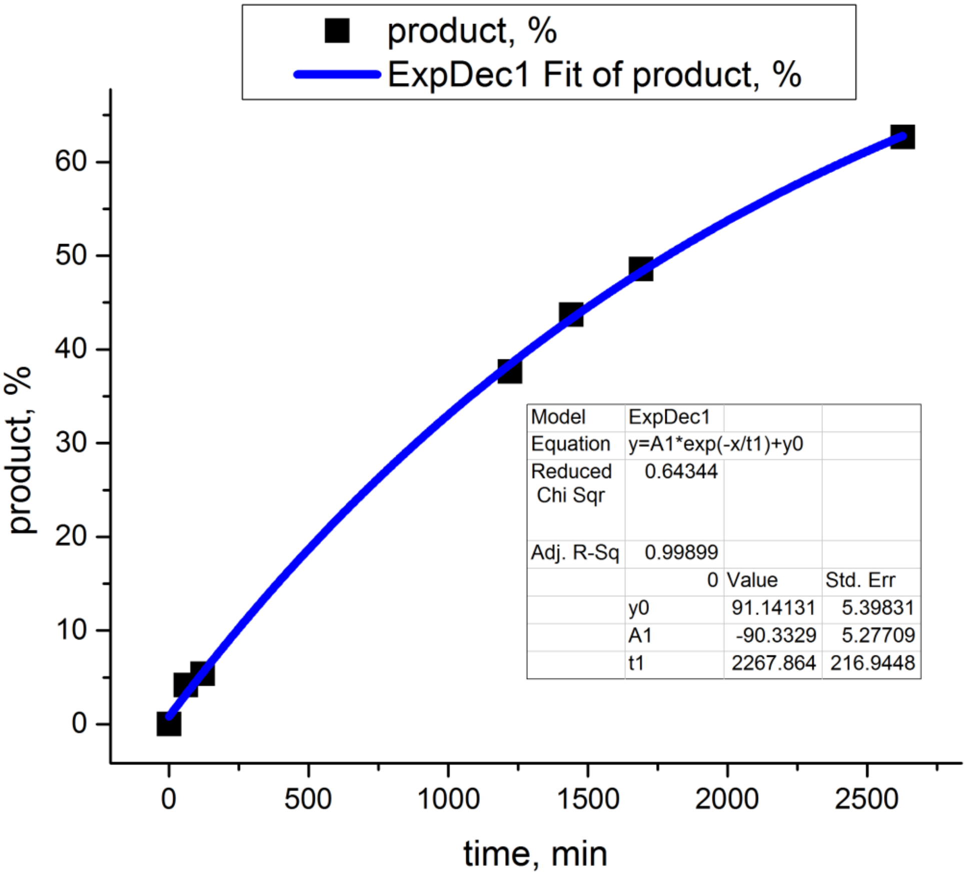

Interpolated $t_{1/2}$: 1783.65

Average $t_{1/2}$: 2056.77
Err $t_{1/2}$[1]: 10.23%
Err log($t_{1/2}$): 0.017

Tetraethylammonium Bromide (TEAB)

Run 1

Time (min)	Standard, μmol	Standard, area	Product, area	Product μmol	Product %
0	263.69	1.00	0.00	0.00	0.00
1	263.69	3935.35	27.59	2.09	0.62
3	263.69	4149.80	91.31	6.57	1.93
5	263.69	4297.15	167.87	11.66	3.43
7	263.69	5558.88	304.61	16.36	4.81
9	263.69	5780.87	417.43	21.55	6.34
15	263.69	2246.00	313.97	41.73	12.27
60	263.69	3318.89	1202.61	108.16	31.81
180	263.69	4298.06	2564.91	178.13	52.39

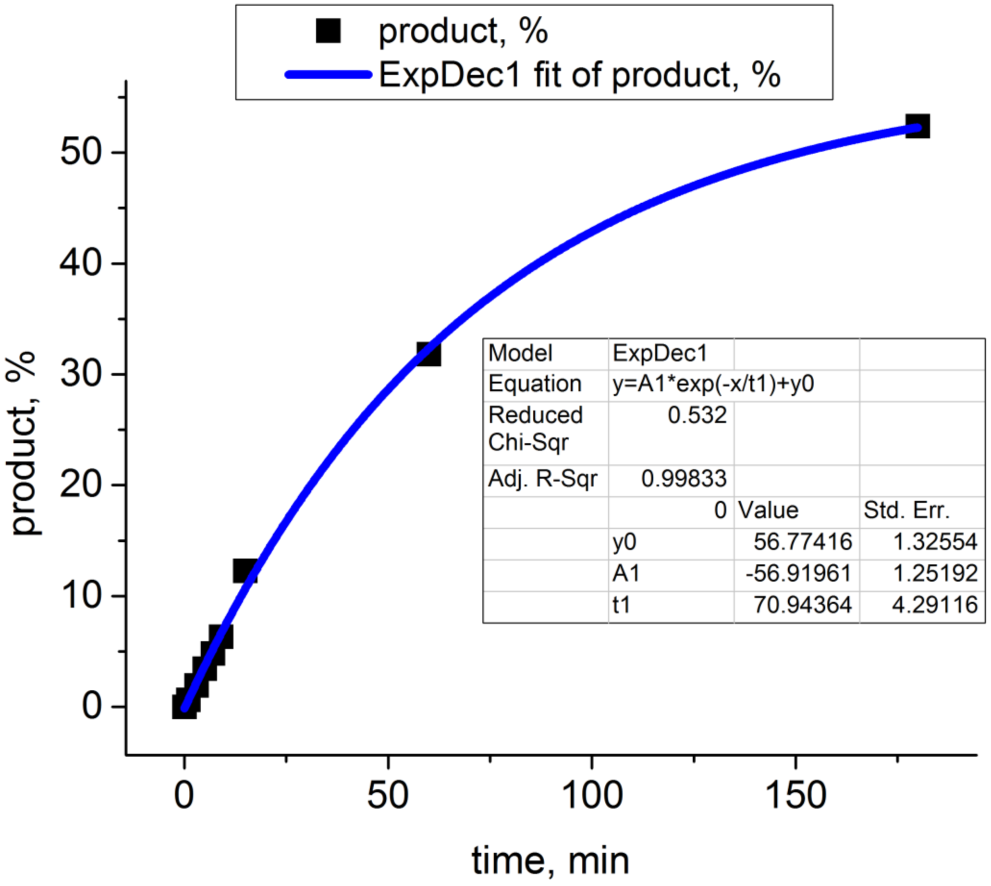

Interpolated $t_{1/2}$: 151.01

Run 2

Time (min)	Standard, µmol	Standard, area	Product, area	Product µmol	Product %
0	263.69	1.00	0.00	0.00	0.00
2	263.69	7466.84	135.94	5.43	1.60
4	263.69	5254.51	202.43	11.50	3.39
6	263.69	3889.61	244.06	18.73	5.53
8	263.69	4478.20	388.41	25.89	7.64
10	263.69	5884.10	587.51	29.80	8.79
16	263.69	4758.59	726.17	45.55	13.44
62	263.69	5012.66	1921.79	114.44	33.77
182	263.69	5216.39	3267.21	186.96	55.16

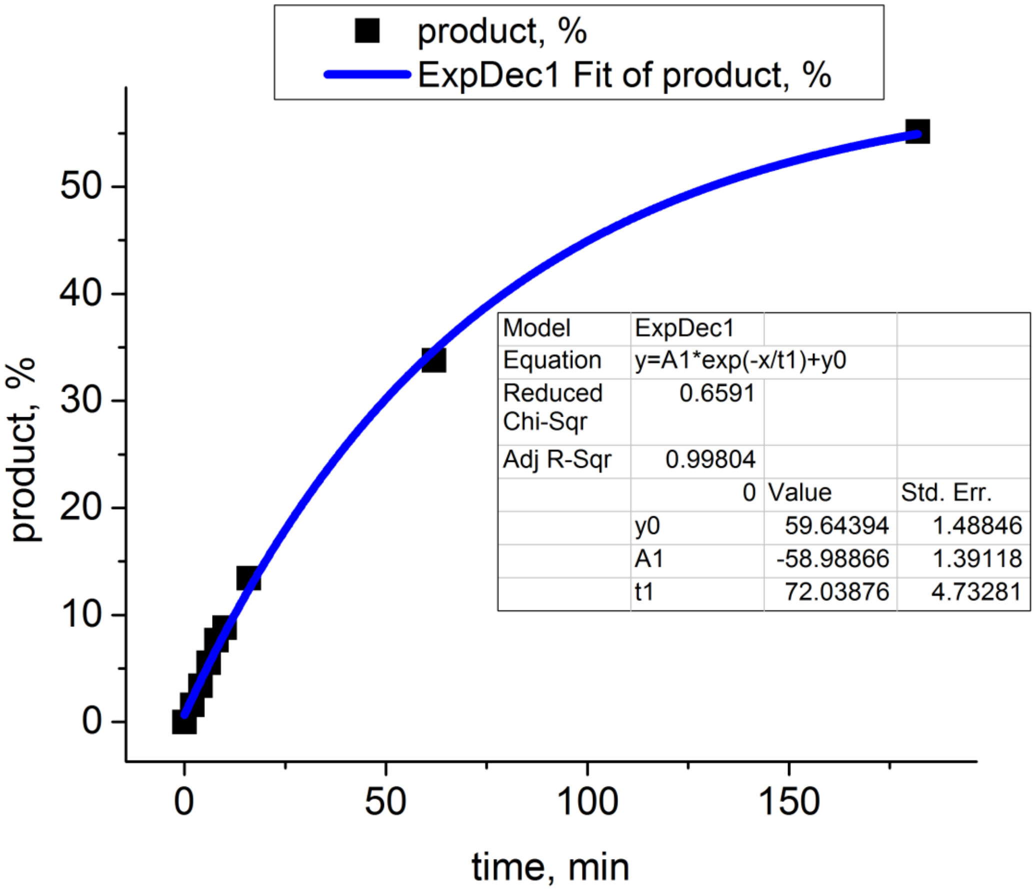

Interpolated $t_{1/2}$: 130.46

Run 3

Time (min)	Standard, µmol	Standard, area	Product, area	Product µmol	Product %
0	263.69	1	0	0.00	0.00
1	263.69	3340.93	25.69	2.29	0.68
3	263.69	5470.12	117.51	6.41	1.89
5	263.69	4158.73	120.00	8.61	2.54
7	263.69	5142.80	255.06	14.80	4.37
9	263.69	3841.22	271.92	21.13	6.24
15	263.69	2883.78	366.42	37.93	11.20
60	263.69	4736.59	1652.10	104.11	30.75
180	263.69	4177.17	2542.23	181.66	53.43

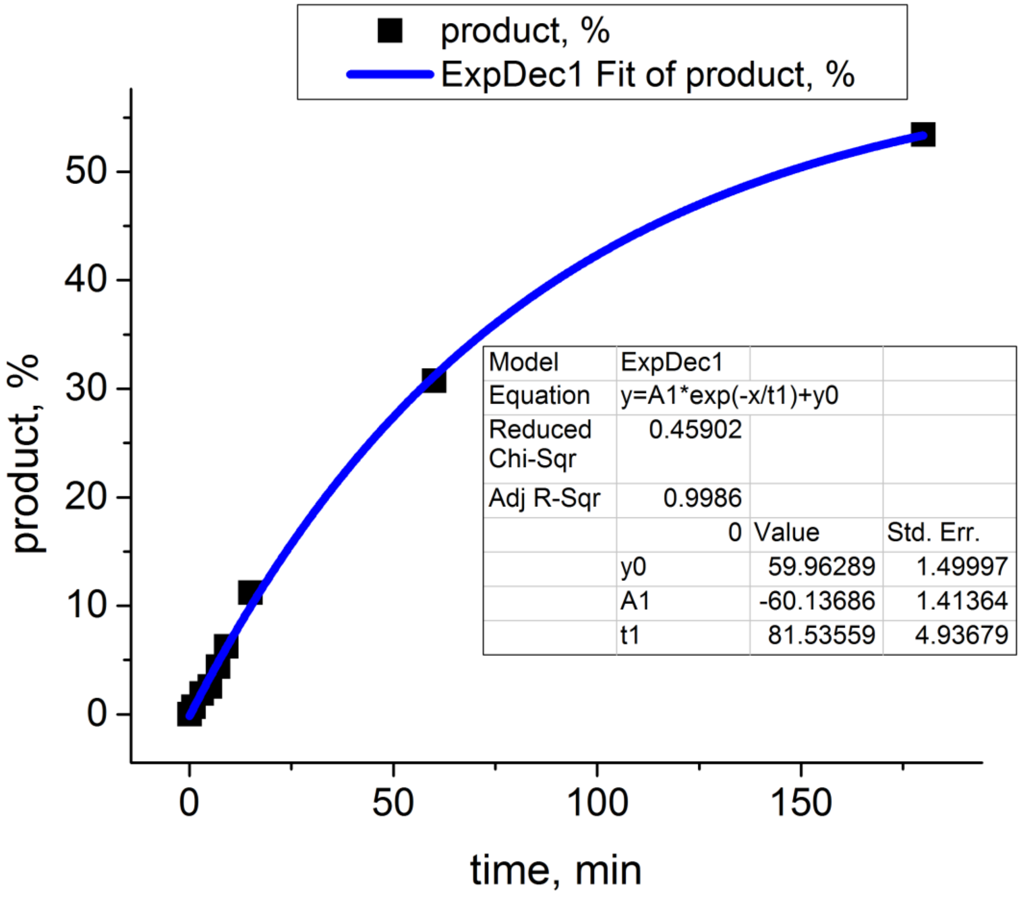

Interpolated $t_{1/2}$: 146.58

Average $t_{1/2}$: 142.68
Err $t_{1/2}$: 6.19%
Err $\log(t_{1/2})$: 0.016

Tetrabutylammonium Bromide (TBAB)

Run 1

Time (min)	Standard, µmol	Standard, area	Product, area	Product µmol	Product %
0	263.69	1.00	0.00	0.00	0.00
1	263.69	5094.16	265.91	15.58	4.53
2	263.69	5086.99	563.14	33.04	9.60
3	263.69	5552.72	868.37	46.68	13.57
4	263.69	5287.03	1075.69	60.73	17.65
5	263.69	4770.05	1200.56	75.13	21.84
6	263.69	4408.55	1323.26	89.59	26.04
7	263.69	5146.33	1769.64	102.64	29.83
9	263.69	5104.58	2296.14	134.27	39.03
10	263.69	6228.27	3126.12	149.82	43.55
15	263.69	5449.60	3961.16	216.97	63.07
30	263.69	5424.35	4870.80	268.03	77.91
60	263.69	5143.59	4737.59	274.93	79.91

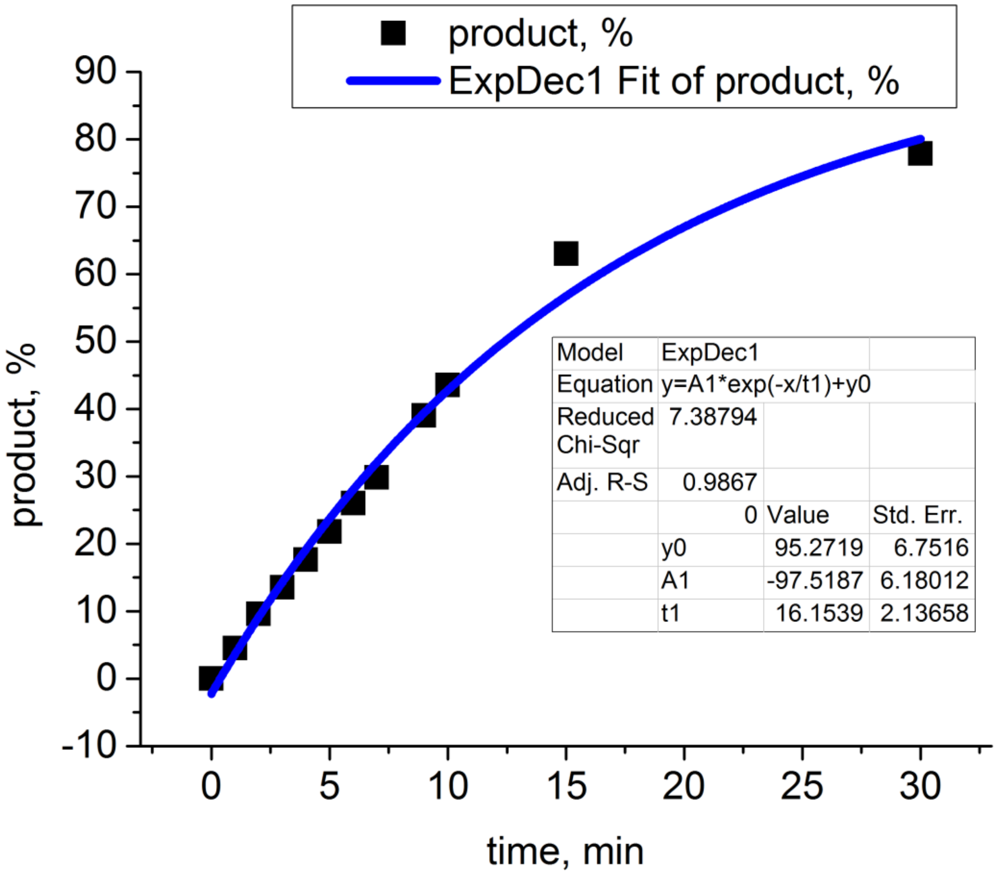

Interpolated $t_{1/2}$: 12.40 min

Run 2

**Time (min)**	**Standard, μmol**	**Standard, area**	**Product, area**	**Product, μmol**	**Product %**
0	263.69	1.00	0.00	0.00	0.00
1	263.69	5295.07	408.82	23.05	6.82
2	263.69	4750.23	562.24	35.33	10.46
3	263.69	4954.93	793.43	47.80	14.15
4	263.69	4179.84	847.82	60.54	17.92
5	263.69	3899.44	976.39	74.74	22.12
6	263.69	4221.56	1232.48	87.14	25.79
7	263.69	4133.84	1372.27	99.09	29.32
8	263.69	3822.86	1421.91	111.02	32.86
9	263.69	5782.25	2391.01	123.43	36.53
10	263.69	5591.14	2520.74	134.57	39.83
15	263.69	3792.12	2467.19	194.20	57.47
30	263.69	5326.97	4769.11	267.23	79.09
62	263.69	4465.91	4046.18	270.44	80.03

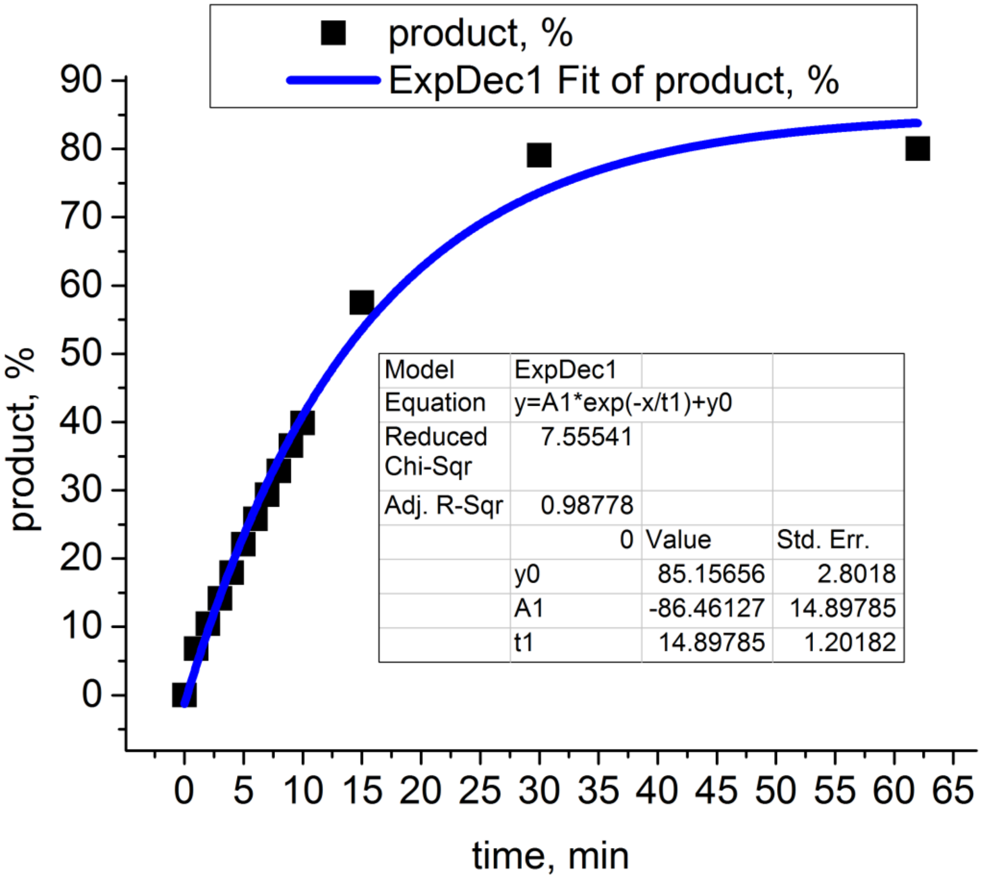

Interpolated $t_{1/2}$: 13.41

Run 3

Time (min)	Standard, µmol	Standard, area	Product, area	Product, µmol	Product %
0	265.73	1.00	0.00	0.00	0.00
1	265.73	5734.28	342.29	17.95	5.29
2	265.73	4151.74	484.81	35.12	10.35
3	265.73	4430.14	725.07	49.23	14.51
5	265.73	4817.95	1213.87	75.78	22.34
6	265.73	4397.58	1276.25	87.30	25.73
7	265.73	4521.87	1476.85	98.24	28.96
8	265.73	4676.11	1689.69	108.69	32.04
9	265.73	4100.21	1608.66	118.01	34.79
10	265.73	5389.11	2318.51	129.41	38.15
15	265.73	3902.23	2193.30	169.07	49.84
30	265.73	4399.70	3392.01	231.90	68.36

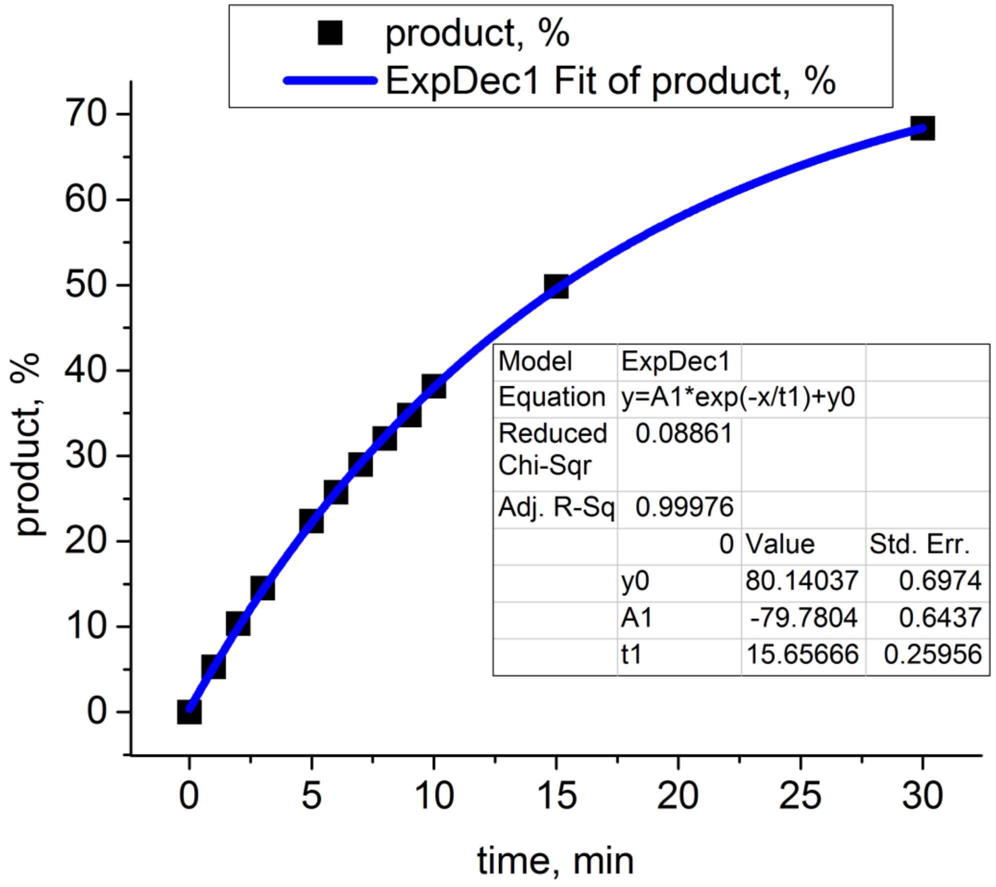

Interpolated $t_{1/2}$: 15.24

Run 4

Time (min)	Standard, µmol	Standard, area	Product, area	Product, µmol	Product %
0	265.73	1.00	0.00	0.00	0.00
1	265.73	5641.42	389.28	20.76	6.11
2	265.73	4732.15	532.46	33.85	9.96
3	265.73	4849.69	749.82	46.51	13.69
4	265.73	3831.83	742.84	58.31	17.16
5	265.73	4037.05	963.57	71.79	21.13
6	265.73	5346.75	1508.33	84.85	24.98
7	265.73	3691.50	1186.49	96.68	28.46
8	265.73	4200.95	1524.34	109.14	32.13
9	265.73	3900.31	1549.18	119.47	35.17
10	265.73	4239.35	1863.67	132.23	38.92
15	265.73	4447.52	2598.06	175.71	51.72
30	265.73	4745.69	3646.99	231.16	68.04

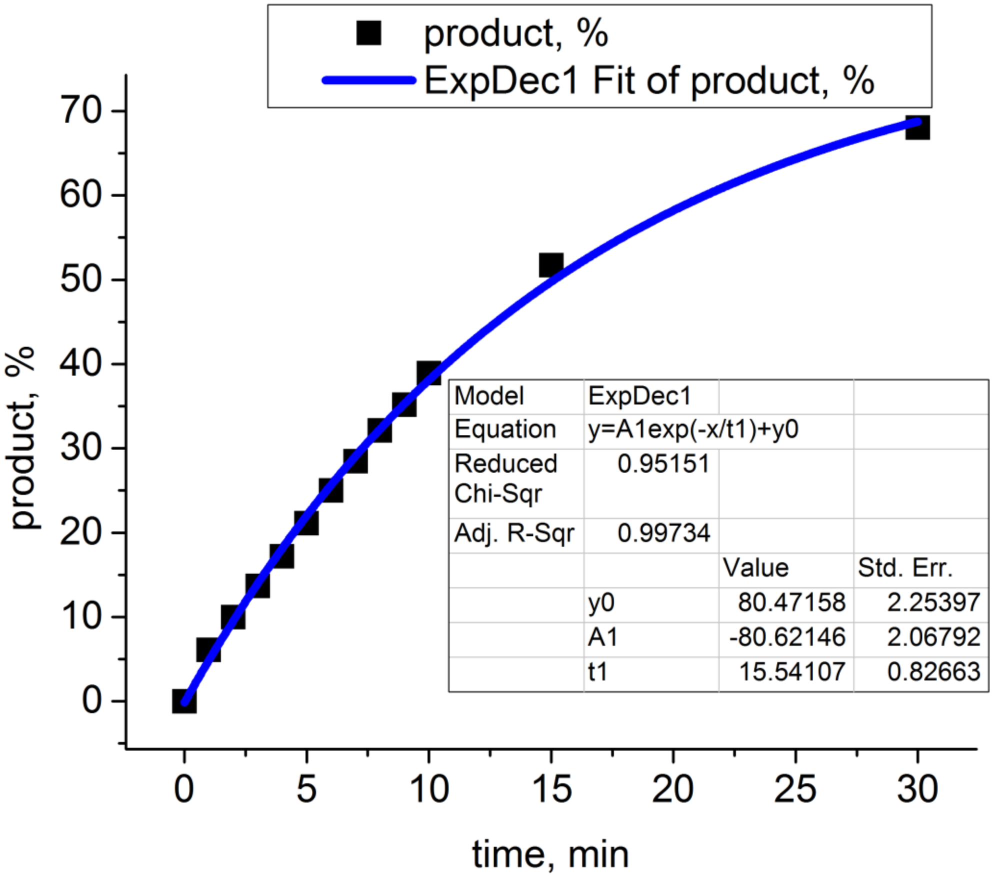

Interpolated $t_{1/2}$: 15.12

Run 5

Time (min)	Standard, µmol	Standard, area	Product, area	Product, µmol	Product %
0	263.69	1.00	0.00	0.00	0.00
1	263.69	4218.09	397.85	28.15	8.30
2	263.69	5791.34	817.68	42.14	12.43
3	263.69	5264.50	1037.11	58.80	17.34
4	263.69	3055.28	724.42	70.77	20.87
5	263.69	3567.16	1004.13	84.02	24.78
6	263.69	3275.18	1073.18	97.81	28.84
7	263.69	3181.30	1155.64	108.43	31.98
8	263.69	5133.85	2170.96	126.22	37.22
9	263.69	4148.73	1888.13	135.85	40.06
10	263.69	3880.56	1881.12	144.70	42.67
15	263.69	5010.21	3097.82	184.56	54.43
30	263.69	4170.00	3123.52	223.58	65.94

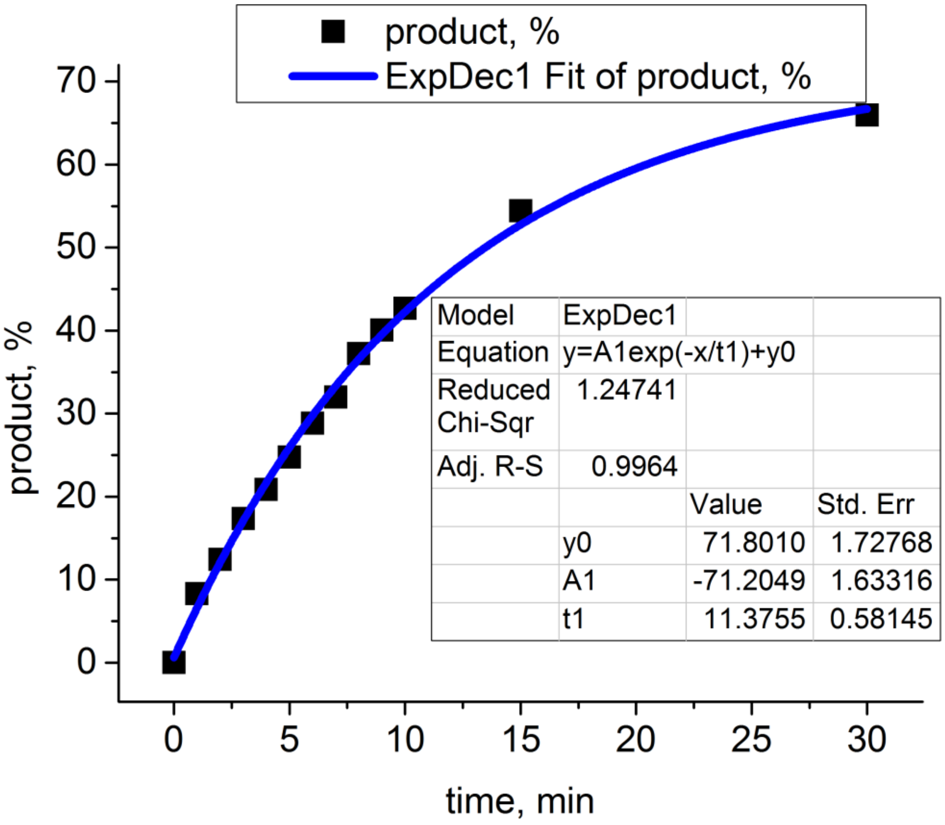

Interpolated $t_{1/2}$: 13.46 min

Average $t_{1/2}$: 13.78 min
Err $t_{1/2}$: 9.42%
Err $\log(t_{1/2})$: 0.030 (3.00%)

Tetrahexylammonium Bromide (THAB)

Run 1

Time (min)	Standard, μmol	Standard, area	Product, area	Product, μmol	Product %
0	263.69	1.00	0.00	0.00	0.00
1	263.69	3519.11	329.38	27.94	8.29
2	263.69	3599.43	351.87	29.18	8.66
3	263.69	4332.65	1170.50	80.64	23.94
4	263.69	4127.63	1457.80	105.42	31.30
5	263.69	5064.61	2086.63	122.98	36.51
6	263.69	3837.72	1722.20	133.95	39.76
7	263.69	4567.27	2671.11	174.57	51.82
8	263.69	5006.19	3163.09	188.60	55.99
9	263.69	3821.01	2497.35	195.09	57.92
10	263.69	3634.86	2430.87	199.62	59.26
15	263.69	4082.23	3101.56	226.79	67.32

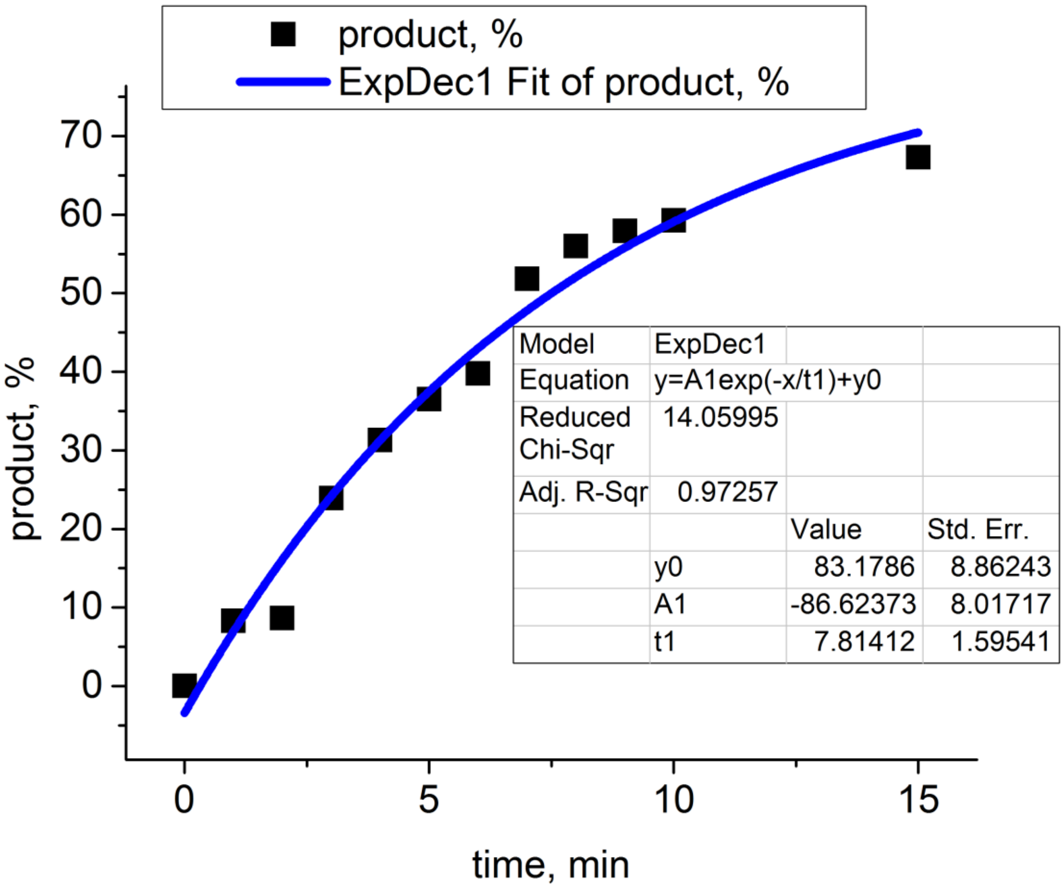

Interpolated $t_{1/2}$: 7.50 min

Run 2

Time (min)	Standard, μmol	Standard, area	Product, area	Product, μmol	Product %
0	263.69	1.00	0.00	0.00	0.00
2	263.69	5867.79	868.70	44.19	12.91
4	263.69	4744.58	1371.84	86.31	25.22
6	263.69	4031.42	1685.30	124.78	36.47
8	263.69	4502.11	2312.98	153.35	44.82
10	263.69	4431.81	2604.50	175.42	51.27
12	263.69	5474.97	3603.12	196.44	57.41
16	263.69	4836.95	3642.97	224.81	65.70

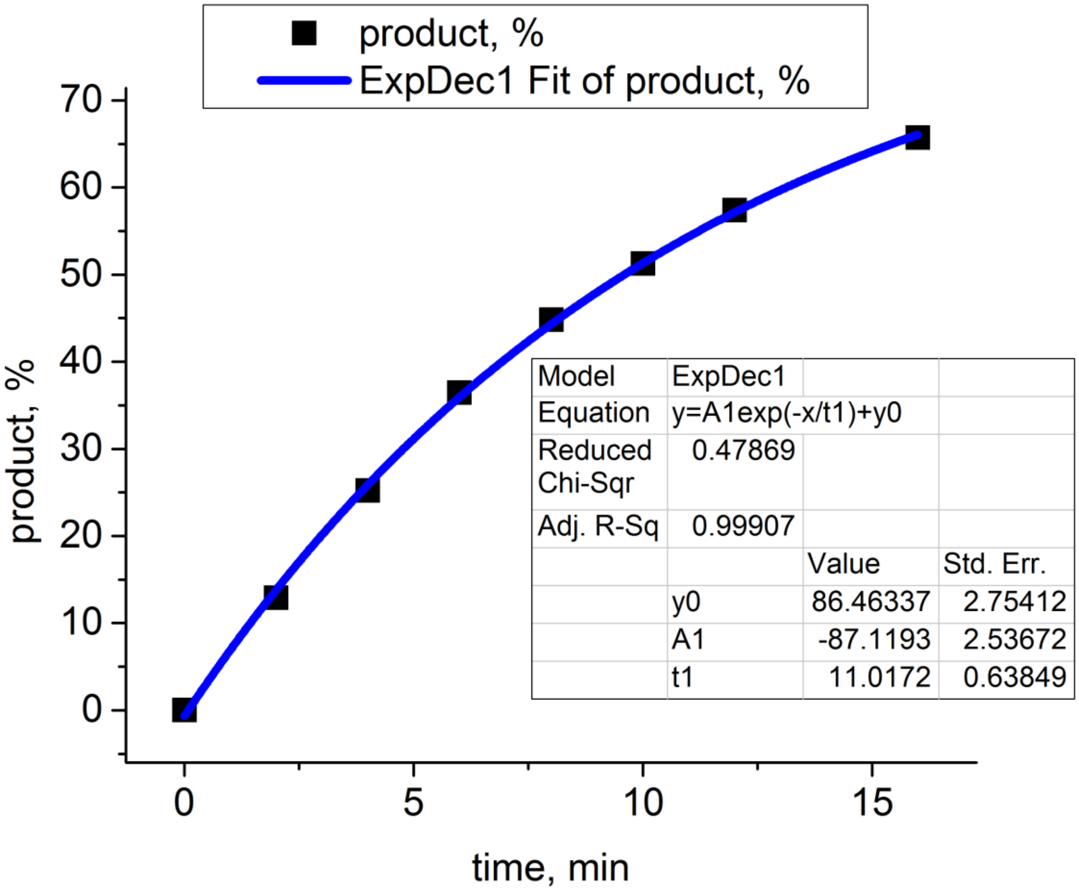

Interpolated $t_{1/2}$: 9.60 min

Run 3

Time (min)	Standard, µmol	Standard, area	Product, area	Product, µmol	Product %
0	263.69	1.00	0.00	0.00	0.00
2	263.69	3623.17	535.27	44.10	12.97
4	263.69	4717.26	1592.61	100.77	29.64
6	263.69	4591.31	2123.74	138.07	40.60
8	263.69	4739.91	2619.07	164.93	48.50
10	263.69	4409.17	2727.46	184.64	54.30
12	263.69	4462.10	2916.58	195.10	57.38
16	263.69	4436.12	3189.66	214.62	63.12

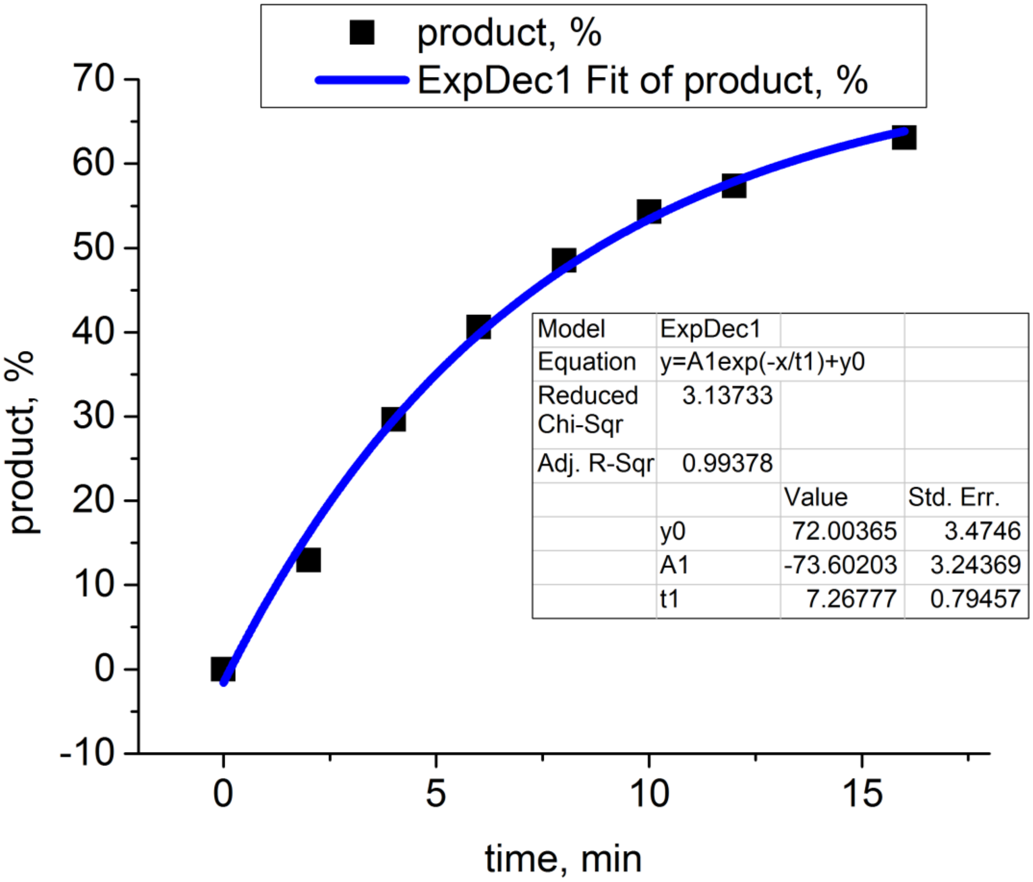

Interpolated $t_{1/2}$: 8.78 min

Run 4

Time (min)	Standard, μmol	Standard, area	Product, area	Product, μmol	Product %
0	263.69	1.00	0.00	0.00	0.00
1	263.69	5175.92	611.51	35.27	10.36
2	263.69	4100.96	741.16	53.95	15.85
3	263.69	4202.38	1004.18	71.33	20.96
4	263.69	4973.19	1469.43	88.20	25.92
5	263.69	4936.57	1715.21	103.71	30.48
6	263.69	8054.49	3388.10	125.56	36.90
7	263.69	7289.25	3361.82	137.67	40.46
8	263.69	5446.21	2659.06	145.74	42.83
9	263.69	3909.09	2036.14	155.48	45.69
10	263.69	4389.88	2479.73	168.61	49.55
15	263.69	4796.84	3520.77	219.09	64.39

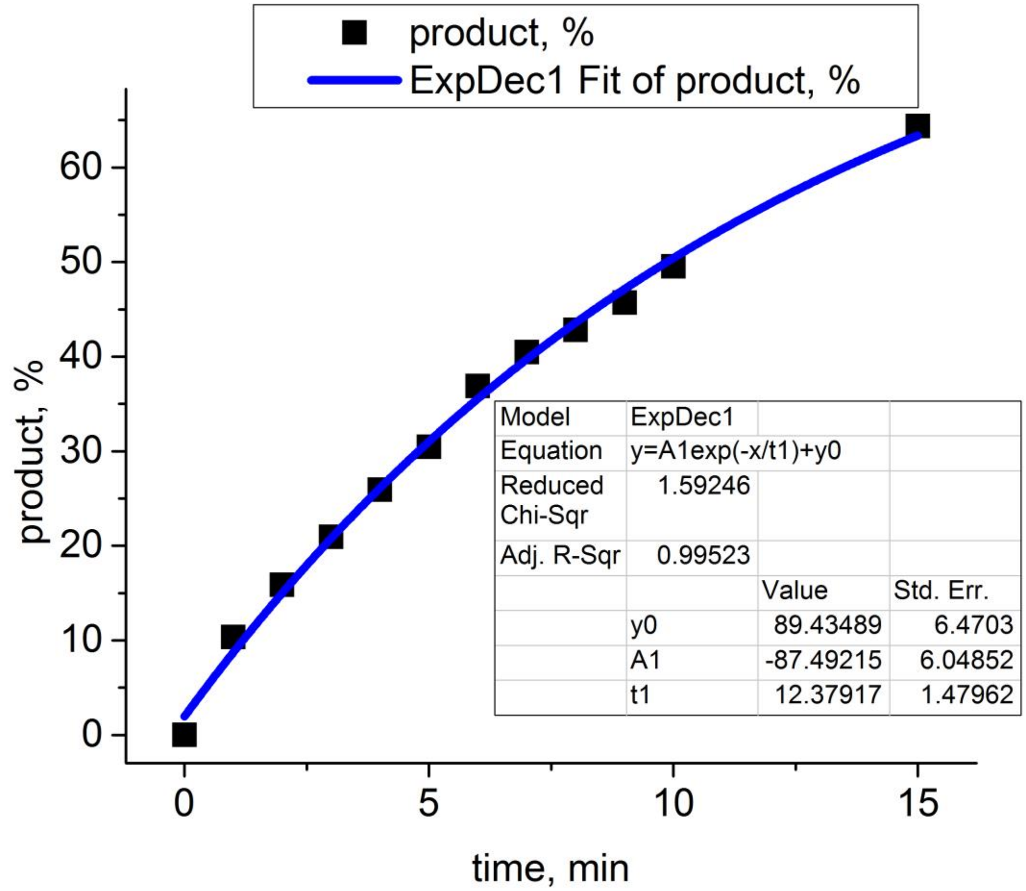

Interpolated $t_{1/2}$: 9.86 min

Run 5

Time (min)	Standard, µmol	Standard, area	Product, area	Product, µmol	Product %
0	263.69	1.00	0.00	0.00	0.00
1	263.69	3381.95	390.15	34.70	10.33
2	263.69	4099.65	757.76	55.60	16.55
3	263.69	4834.43	1203.26	74.87	22.29
4	263.69	4444.84	1371.02	92.78	27.62
5	263.69	5592.06	2037.49	109.60	32.63
6	263.69	3763.52	1522.01	121.64	36.21
7	263.69	4408.61	1978.92	135.02	40.20
8	263.69	4486.56	2215.22	148.52	44.21
9	263.69	4230.66	2230.47	158.58	47.21
10	263.69	5018.22	2819.27	168.99	50.31
15	263.69	5106.78	3973.17	234.02	69.67

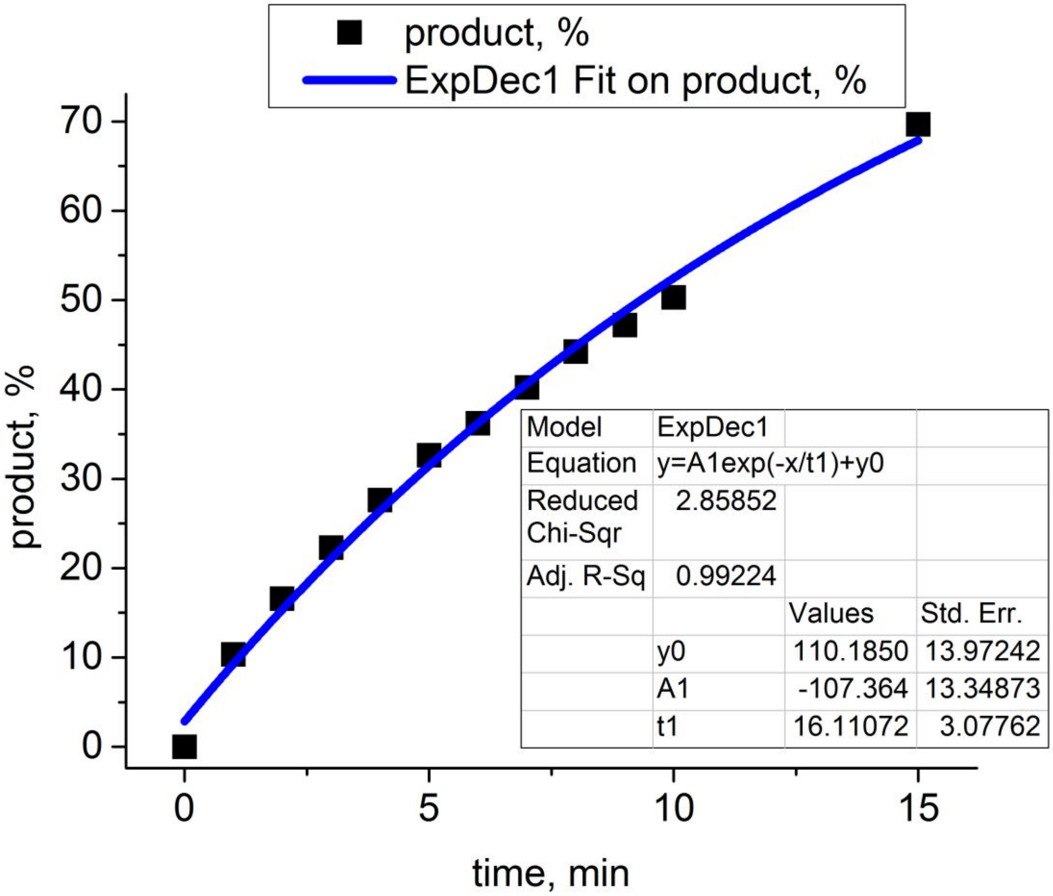

Interpolated $t_{1/2}$: 9.32 min

Average $t_{1/2}$: 9.01 min
Err $t_{1/2}$: 9.30%
Err $\log(t_{1/2})$: 0.045

Tetraheptylammonium Bromide (THepAB)

The larger variability in the t1/2 data for THepAB arises from a tendency to emulsify biphasic mixture. Run 1

Time (min)	Standard, μmol	Standard, area	Product, area	Product, μmol	Product %
0	263.69	1.00	0.00	0.00	0.00
1	263.69	2886.30	213.70	22.10	6.51
2	263.69	5086.30	652.90	38.32	11.28
3	263.69	4207.40	785.40	55.72	16.41
4	263.69	3715.00	896.50	72.03	21.21
5	263.69	5282.80	1546.00	87.35	25.72
6	263.69	5525.80	1915.40	103.47	30.46
7	263.69	5052.90	1992.00	117.67	34.65
8	263.69	5223.40	2280.80	130.34	38.38
9	263.69	4707.30	2282.90	144.76	42.62
10	263.69	3744.50	1937.30	154.43	45.47
15	263.69	4972.50	3412.30	204.84	60.31

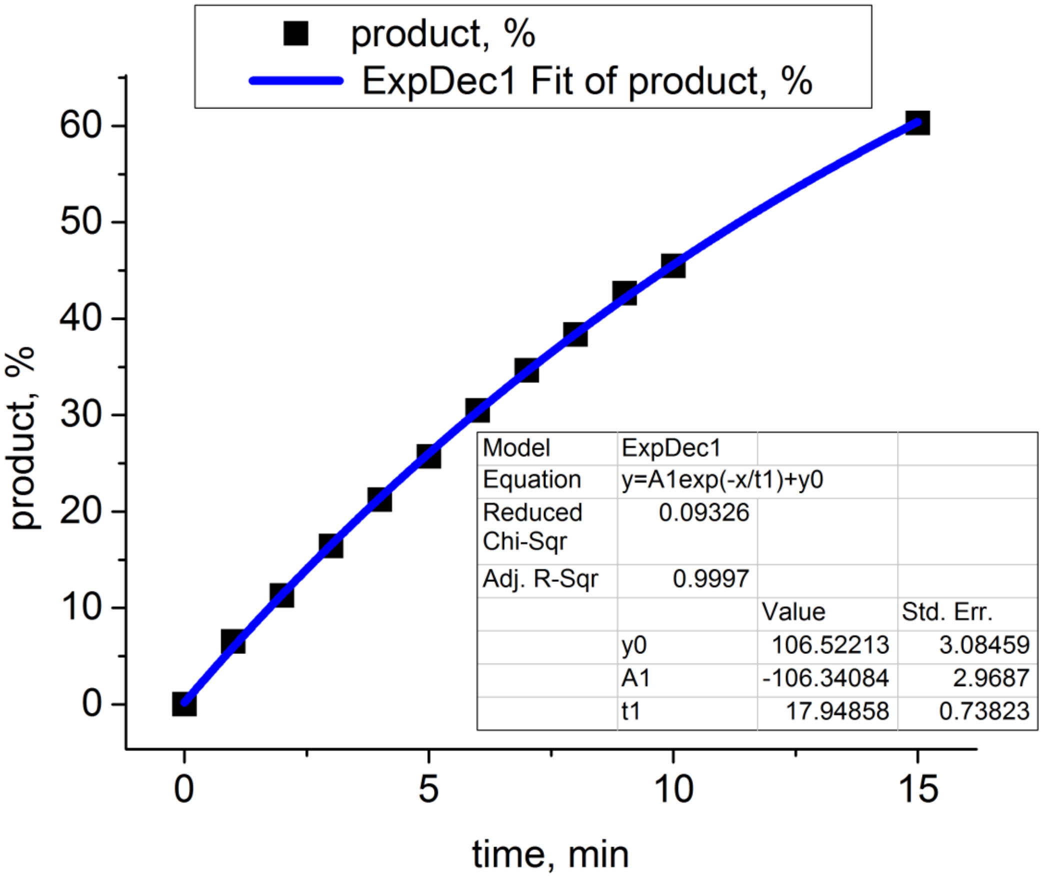

Interpolated $t_{1/2}$: 11.34 min

Run 2

Time (min)	Standard, μmol	Standard, area	Product, area	Product, μmol	Product %
0	263.69	1.00	0.00	0.00	0.00
1	263.69	4625.30	489.00	31.56	9.30
2	263.69	3709.40	638.60	51.39	15.14
3	263.69	3800.60	899.10	70.61	20.80
4	263.69	4179.00	1230.10	87.86	25.89
5	263.69	5155.30	1819.10	105.33	31.03
6	263.69	4674.20	1889.00	120.63	35.54
7	263.69	4522.50	2009.10	132.60	39.07
8	263.69	5669.80	2800.60	147.44	43.44
9	263.69	4418.30	2360.00	159.44	46.97
10	263.69	5707.90	3290.00	172.05	50.69
15	263.69	4903.70	3739.00	227.60	67.05

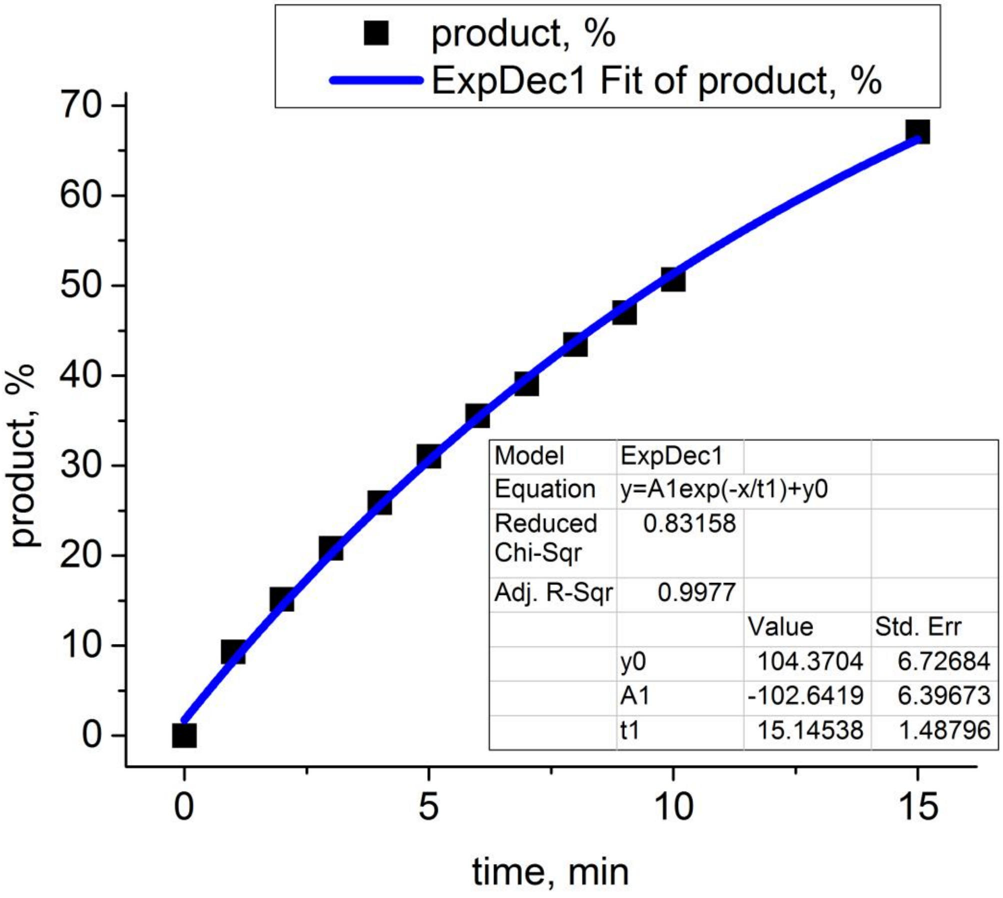

Interpolated $t_{1/2}$: 9.62 min

Run 3

Time (min)	Standard, μmol	Standard, area	Product, area	Product, μmol	Product %
0	263.69	1.00	0.00	0.00	0.00
1	263.69	4256.50	457.20	32.06	9.46
2	263.69	3050.80	646.30	63.23	18.65
3	263.69	5021.10	1588.40	94.43	27.85
4	263.69	6027.70	2469.60	122.29	36.07
5	263.69	5231.50	2409.70	137.49	40.55
6	263.69	1662.80	811.40	145.66	42.96
7	263.69	5065.60	2790.90	164.45	48.50
8	263.69	4660.40	2787.80	178.55	52.66
9	263.69	5799.70	3590.70	184.80	54.51
10	263.69	4542.70	2925.00	192.20	56.69
15	263.69	6135.10	4211.50	204.90	60.43

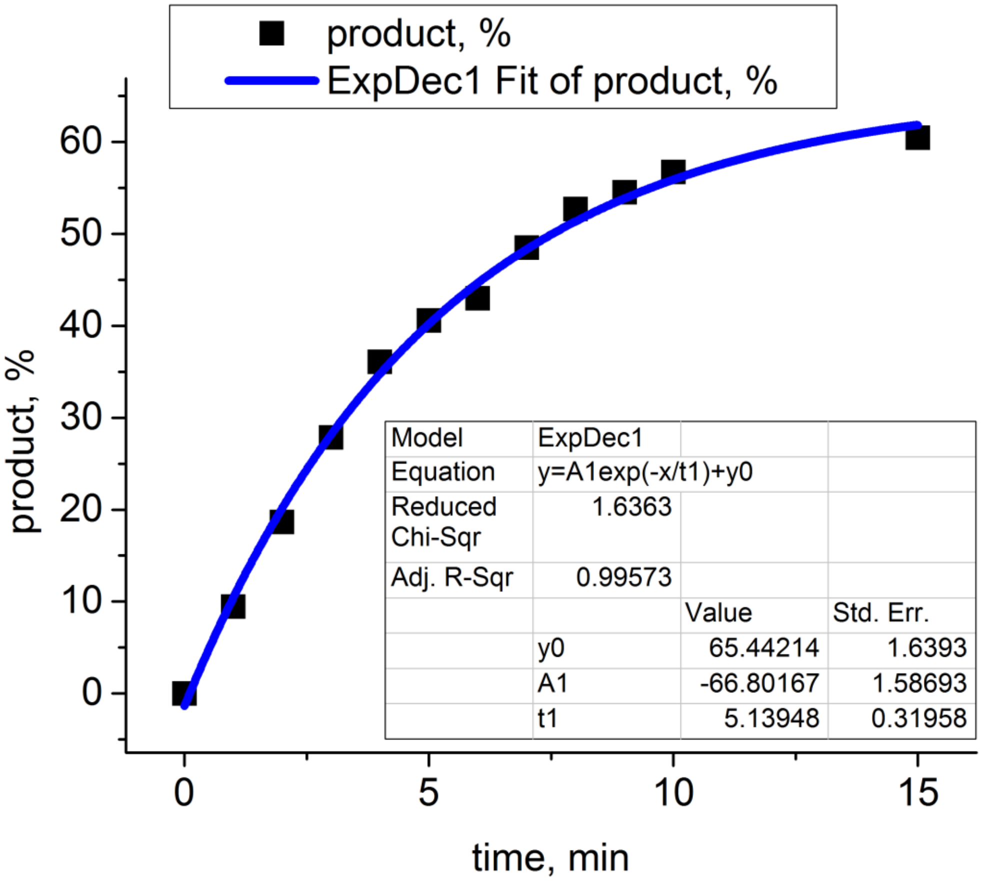

Interpolated $t_{1/2}$: 7.53 min

Average $t_{1/2}$: 9.50 min
Err $t_{1/2}$: 16.4%
Err $\log(t_{1/2})$: 0.075

Tetraoctylammonium Bromide (TOAB)

Run 1

Time (min)	Standard, μmol	Standard, area	Product, area	Product, μmol	Product %
0	263.69	1.00	0.00	0.00	0.00
1	263.69	5001.26	532.04	31.75	9.07
2	263.69	4133.34	745.08	53.81	15.37
3	263.69	5615.28	1469.20	78.10	22.31
4	263.69	4725.28	1569.81	99.16	28.33
5	263.69	4596.13	1767.24	114.77	32.79
6	263.69	5750.63	2554.45	132.59	37.88
7	263.69	5383.74	2591.72	143.69	41.05
8	263.69	6281.86	3293.46	156.49	44.71
9	263.69	6072.91	3380.25	166.14	47.46
10	263.69	4877.12	2831.03	173.27	49.50
15	263.69	4711.05	3342.22	211.76	60.49

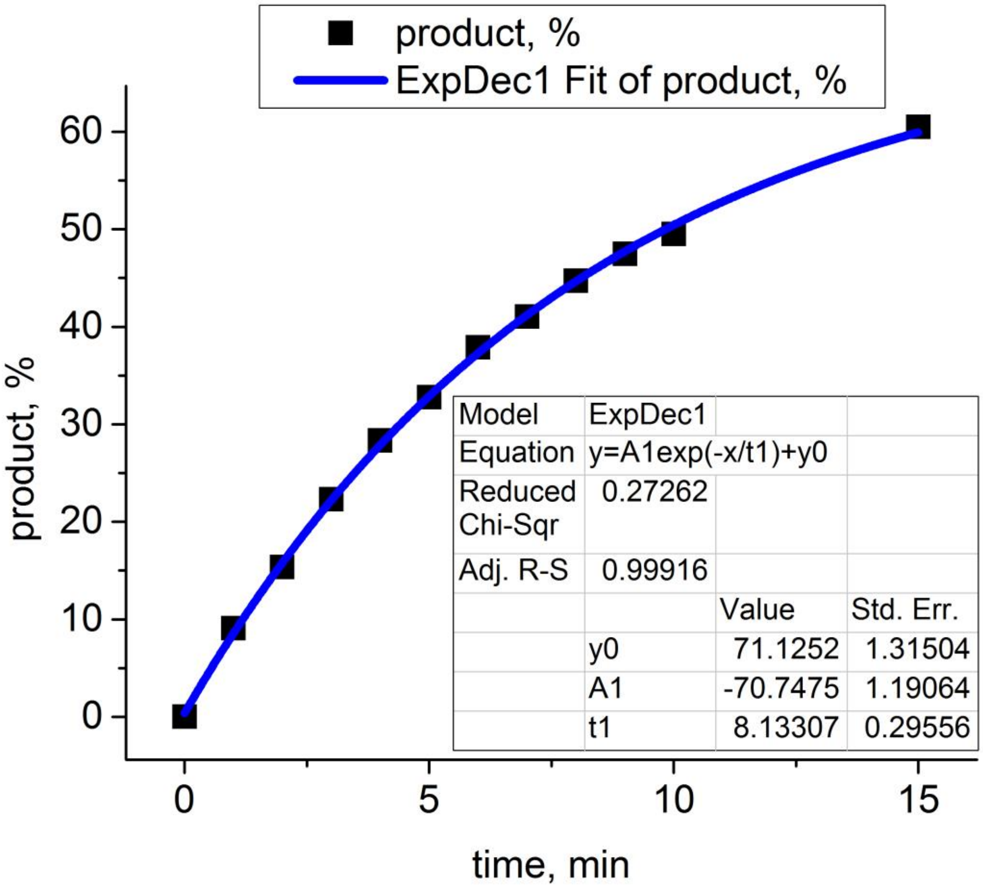

Interpolated $t_{1/2}$: 9.83 min

Run 2

Time (min)	Standard, μmol	Standard, area	Product, area	Product, μmol	Product %
0	263.69	1.00	0.00	0.00	0.00
1	263.69	5643.99	730.85	38.65	11.40
2	263.69	6648.15	1263.88	56.75	16.74
3	263.69	5238.13	1234.85	70.37	20.76
4	263.69	5594.03	1563.20	83.41	24.61
5	263.69	6167.95	2043.68	98.90	29.18
6	263.69	4765.79	1730.91	108.41	31.98
7	263.69	5233.26	2106.31	120.14	35.44
8	263.69	5191.13	2246.02	129.15	38.10
9	263.69	4026.09	1825.68	135.36	39.93
10	263.69	8262.06	4314.44	155.87	45.99
15	263.69	4060.10	2564.52	188.54	55.62
30	263.69	5975.69	4293.91	214.49	63.28

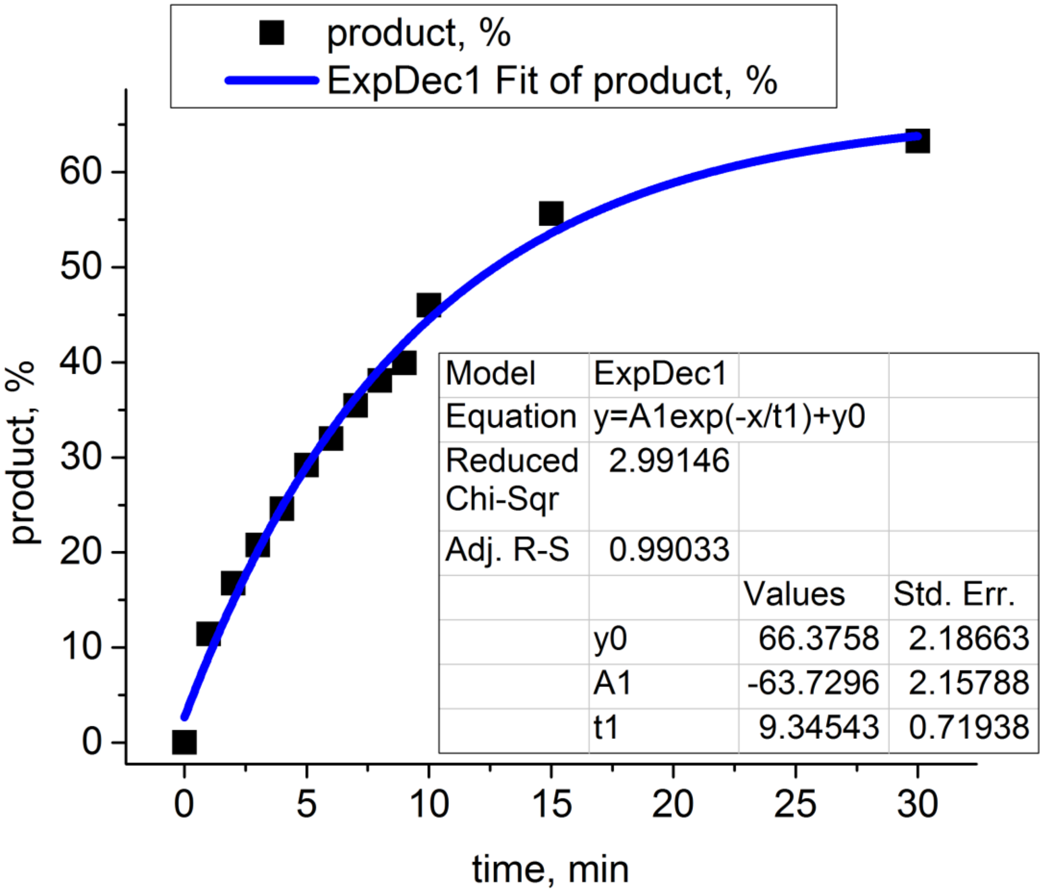

Interpolated $t_{1/2}$: 12.70 min

Run 3

Time (min)	Standard, µmol	Standard, area	Product, area	Product, µmol	Product %
0	263.69	1.00	0.00	0.00	0.00
1	263.69	4698.86	443.63	28.18	8.30
2	263.69	7297.82	1088.46	44.52	13.10
3	263.69	3971.99	794.15	59.68	17.57
4	263.69	5696.84	1457.85	76.39	22.48
5	263.69	7682.39	2316.03	89.99	26.49
6	263.69	4139.96	1384.18	99.80	29.38
7	263.69	5264.42	1996.19	113.18	33.32
8	263.69	6486.81	2773.01	127.60	37.56
9	263.69	4547.12	2021.86	132.72	39.07
10	263.69	4934.31	2381.88	144.09	42.41
15	263.69	4419.48	2756.01	186.14	54.79
30	263.69	4292.90	3036.26	211.12	62.14

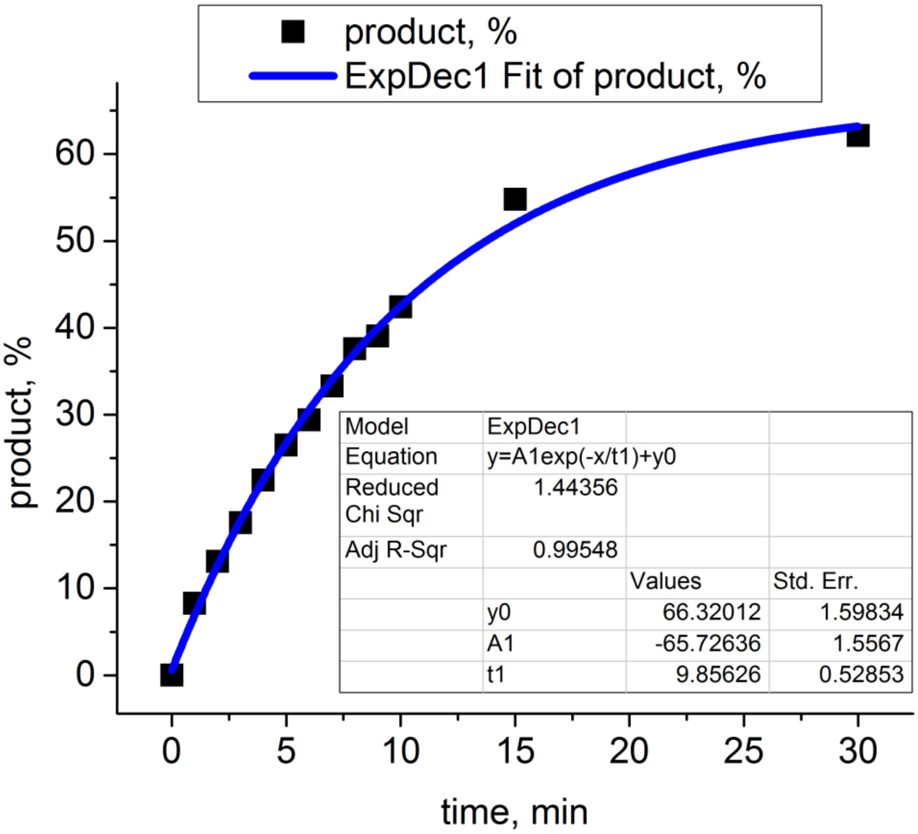

Interpolated $t_{1/2}$: 13.73 min

Average $t_{1/2}$:12.09 min
Err $t_{1/2}$:13.7%
Err log($t_{1/2}$): 0.058

Tetradecylammonium Bromide (TDAB)

Run 1

Time (min)	Standard, μmol	Standard, area	Product, area	Product, μmol	Product %
0	263.69	1.00	0.00	0.00	0.00
1	263.69	4353.32	44.51	3.05	0.90
2	263.69	5421.08	117.94	6.49	1.92
3	263.69	5603.12	182.23	9.71	2.86
4	263.69	6033.57	256.62	12.70	3.75
5	263.69	8044.60	439.77	16.32	4.82
6	263.69	6004.68	390.12	19.39	5.72
7	263.69	5043.30	384.89	22.78	6.72
8	263.69	4816.70	430.55	26.68	7.87
9	263.69	5294.88	551.74	31.10	9.18
10	263.69	5629.92	665.66	35.29	10.42
15	263.69	4764.60	948.36	59.41	17.53
30	263.69	5998.56	2214.56	110.20	32.52
60	263.69	5230.10	2830.40	161.54	47.67
120	263.69	4580.96	2950.01	192.22	56.73

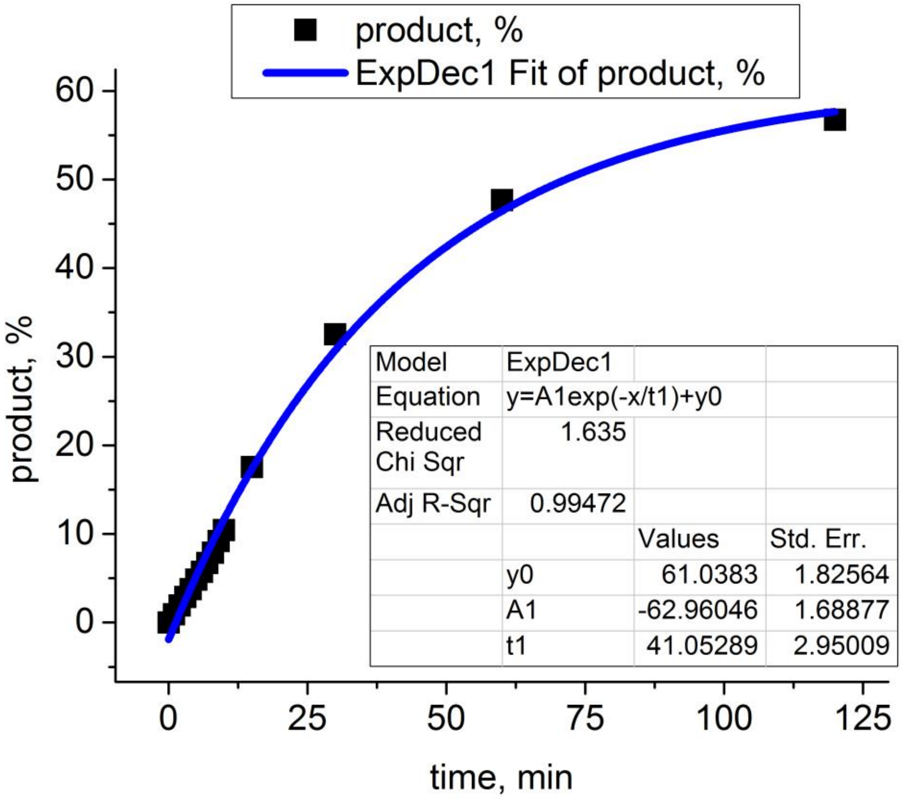

Interpolated $t_{1/2}$: 71.48 min

Run 2

Time (min)	Standard, µmol	Standard, area	Product, area	Product, µmol	Product %
0	263.69	1.00	0.00	0.00	0.00
1	263.69	5116.19	20.87	1.22	0.36
2	263.69	4108.11	67.64	4.92	1.44
3	263.69	4416.88	130.48	8.82	2.59
4	263.69	4752.25	193.23	12.14	3.56
5	263.69	6378.95	323.18	15.12	4.43
6	263.69	4736.70	277.50	17.49	5.13
7	263.69	6335.92	438.16	20.64	6.05
8	263.69	7829.19	623.93	23.79	6.97
9	263.69	5269.09	465.23	26.36	7.73
10	263.69	5873.27	574.43	29.19	8.56
15	263.69	4126.10	614.06	44.42	13.02
30	263.69	5367.76	1580.20	87.87	25.76
60	263.69	4022.66	1858.54	137.91	40.43
120	263.69	5569.08	3519.84	188.66	55.31

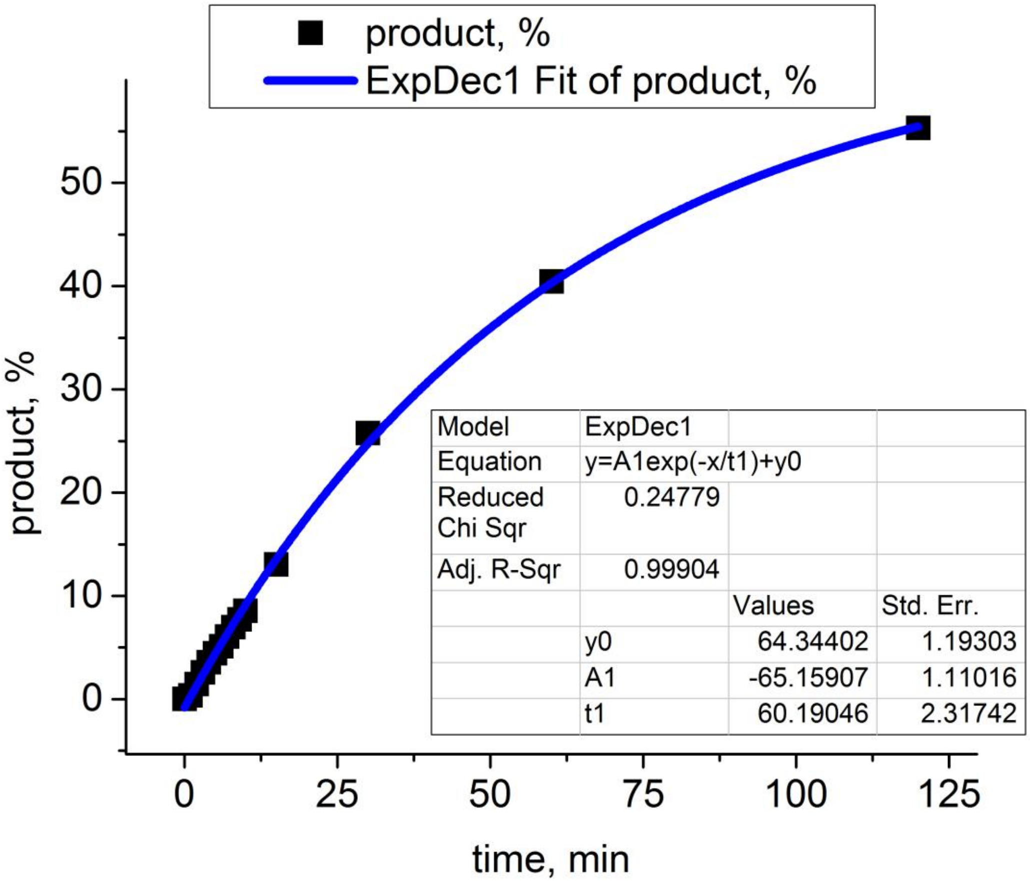

Interpolated $t_{1/2}$: 91.10 min

Run 3

Time (min)	Standard, μmol	Standard, area	Product, area	Product, μmol	Product %
0	263.69	1.00	0.00	0.00	0.00
1	263.69	5896.04	43.28	2.19	0.65
2	263.69	5004.65	104.09	6.21	1.84
3	263.69	4911.40	157.06	9.55	2.82
4	263.69	4910.62	213.03	12.95	3.83
5	263.69	5757.56	306.58	15.89	4.70
6	263.69	5328.70	330.31	18.50	5.48
7	263.69	4693.03	333.31	21.20	6.27
8	263.69	10159.70	847.34	24.89	7.37
10	263.69	8411.82	844.29	29.96	8.87
15	263.69	6243.57	971.85	46.46	13.75
30	263.69	6019.80	1843.11	91.39	27.05
60	263.69	3620.34	1773.55	146.23	43.27
120	263.69	5032.03	3242.49	192.34	56.92

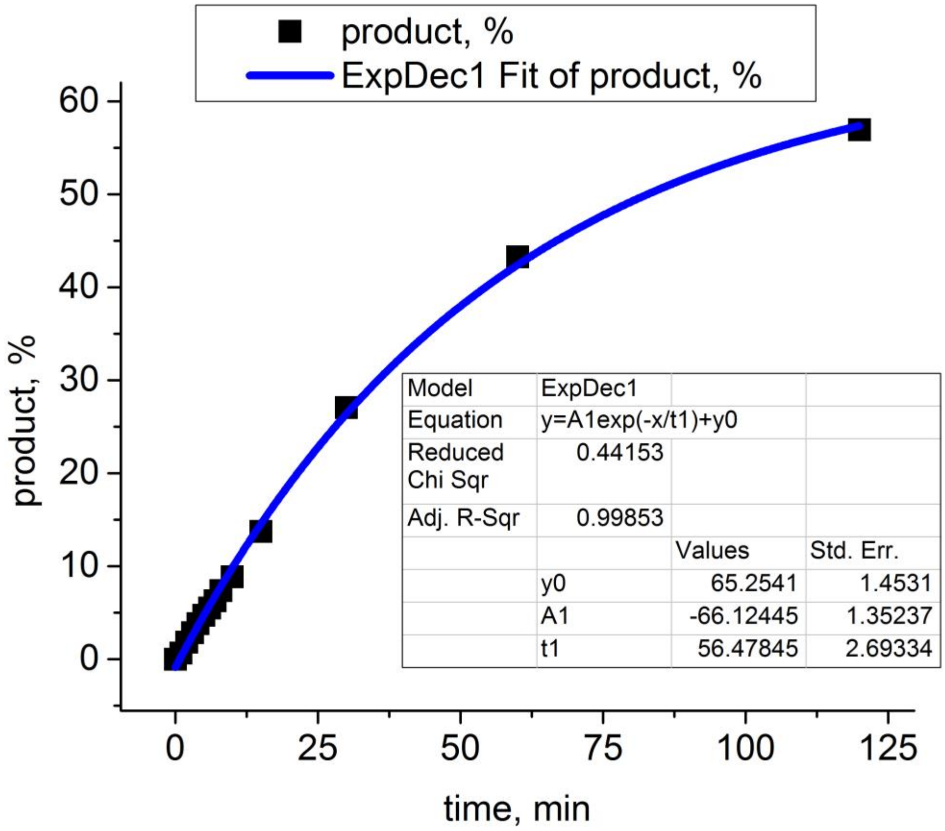

Interpolated $t_{1/2}$: 82.84 min

Average $t_{1/2}$:81.80 min
Err $t_{1/2}$: 9.83%
Err $\log(t_{1/2})$: 0.023

Tetradodecylammonium Bromide (TDoDAB)

Run 1

Time (min)	Standard, μmol	Standard, area	Product, area	Product, μmol	Product %
0	263.69	1.00	0.00	0.00	0.00
60	263.69	4122.35	859.58	62.24	18.27
120	263.69	4872.01	1444.34	88.49	25.97
240	263.69	4027.17	1746.44	129.45	37.99
420	263.69	4057.66	2331.48	171.51	50.34
540	263.69	4787.82	3087.00	192.46	56.49

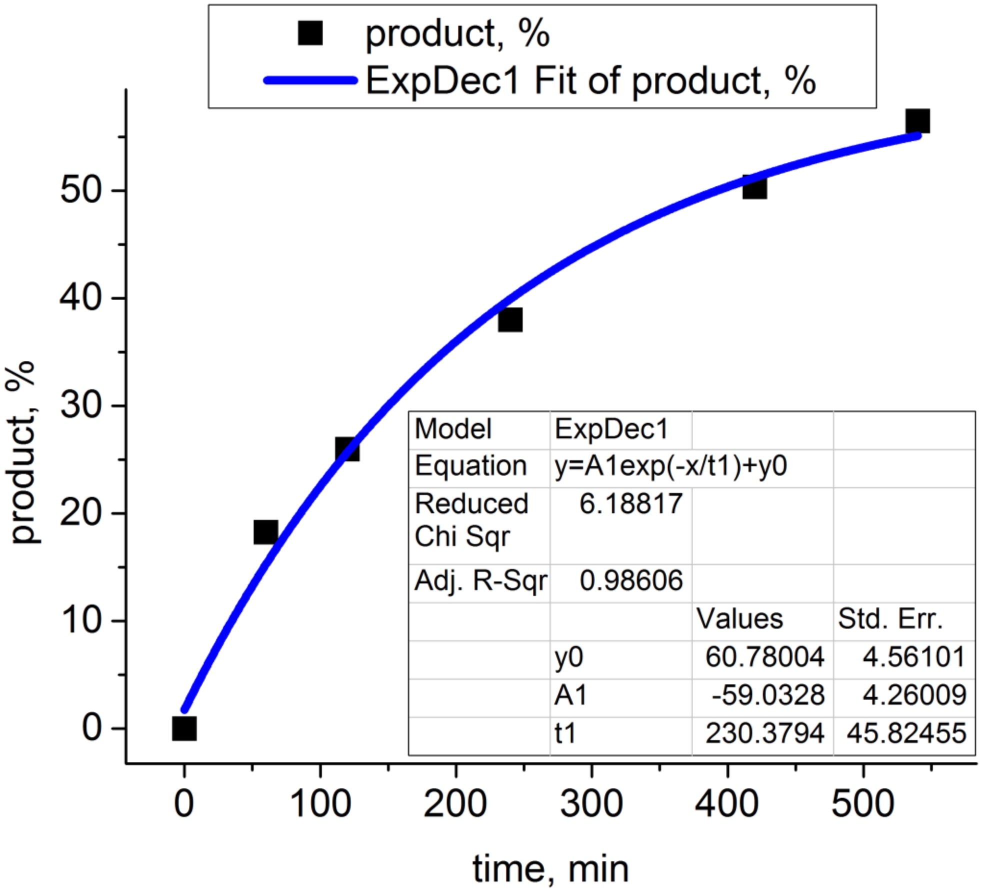

Interpolated $t_{1/2}$: 391.74 min

Run 2

Time (min)	Standard, μmol	Standard, area	Product, area	Product, μmol	Product %
0	263.69	1.00	0.00	0.00	0.00
60	263.69	4474.42	768.25	51.25	15.06
120	263.69	3827.86	915.60	71.40	20.98
240	263.69	4211.02	1763.19	124.98	36.73
420	263.69	3094.14	1716.65	165.61	48.66
540	263.69	5132.79	3271.39	190.24	55.90

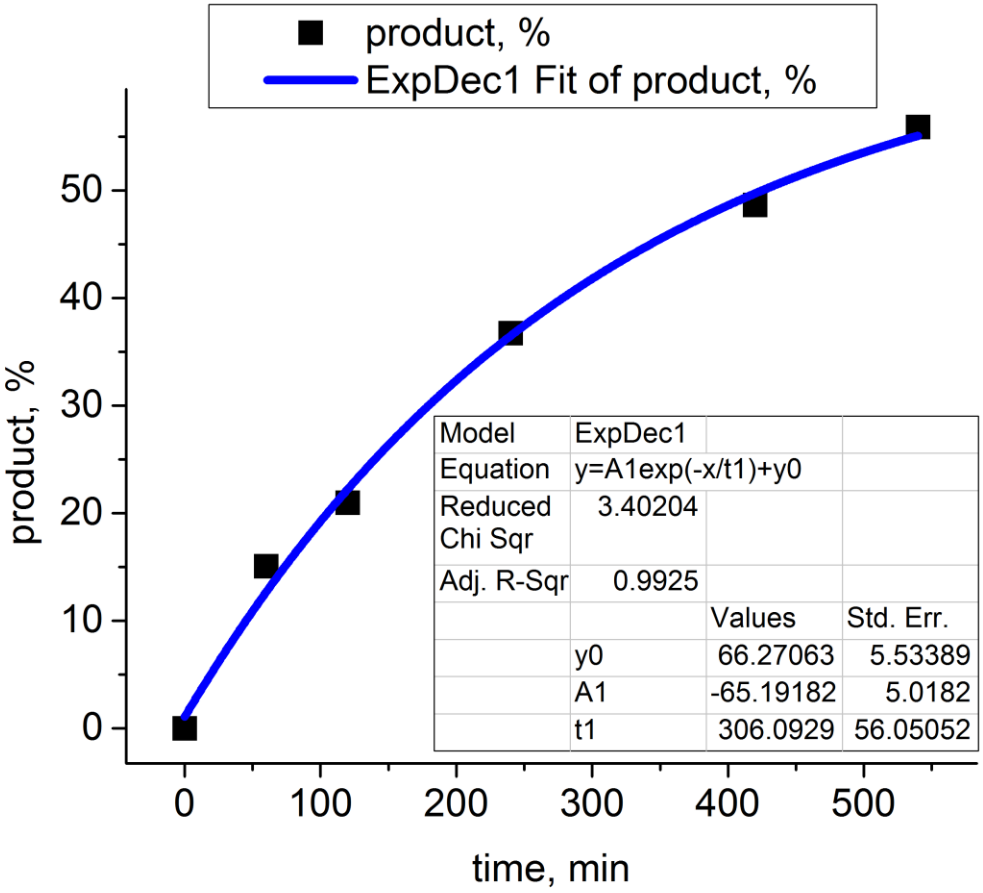

Interpolated $t_{1/2}$: 424.85 min

Run 3

Time (min)	Standard, μmol	Standard, area	Product, area	Product, μmol	Product %
0	263.69	1.00	0.00	0.00	0.00
60	263.69	4169.68	885.16	63.37	18.48
120	263.69	4877.51	1446.53	88.52	25.81
240	263.69	4000.58	1735.62	129.50	37.76
420	263.69	4171.94	2306.29	165.01	48.12
540	263.69	4904.78	3118.22	189.77	55.33

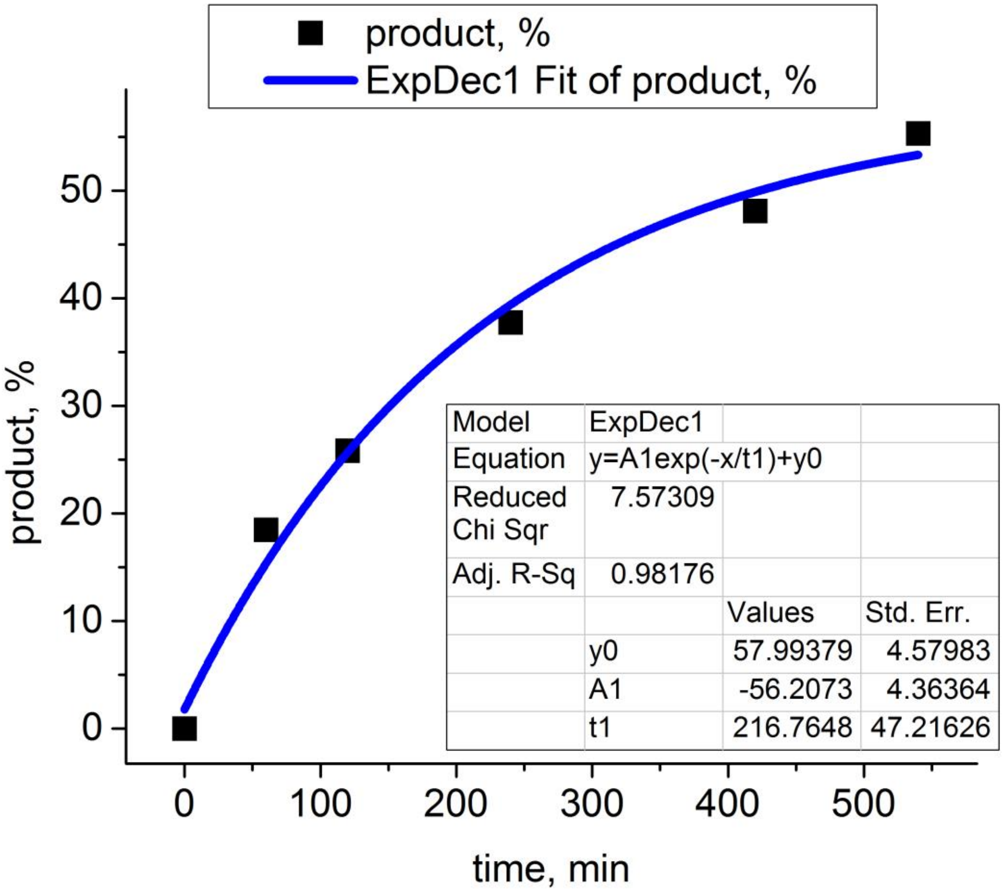

Interpolated $t_{1/2}$: 422.78 min

Average $t_{1/2}$: 413.12 min
Err $t_{1/2}$: 3.67%
Err $\log(t_{1/2})$: 0.0061

Tetrakis(hexadecyl)ammonium bromide (THexDAB)

Run 1

Time (min)	Standard, μmol	Standard, area	Product, area	Product, μmol	Product %
0	263.69	1.00	0.00	0.00	0.00
15	263.69	4510.90	14.39	0.95	0.28
30	263.69	5783.30	39.88	2.06	0.61
60	263.69	5486.59	72.69	3.95	1.16
130	263.69	6298.57	106.59	5.05	1.49
240	263.69	4716.78	134.12	8.49	2.50
360	263.69	6787.08	288.34	12.68	3.73
480	263.69	6098.58	402.22	19.69	5.79
1394	263.69	7038.64	1317.32	55.86	16.44
1830	263.69	5966.81	1396.69	69.87	20.56
2914	263.69	7272.89	2578.55	105.83	31.15
5796	263.69	5977.35	3565.85	178.07	52.41

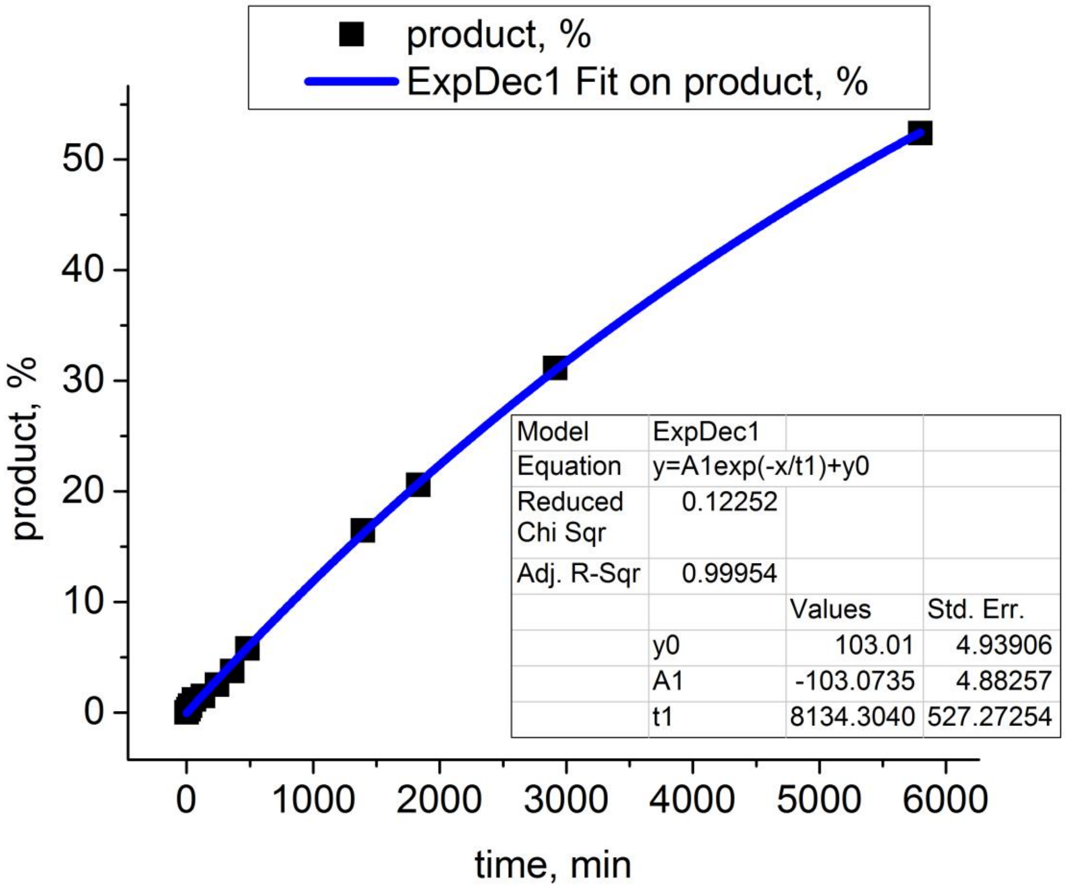

Interpolated $t_{1/2}$: 5409.00

Run 2

Time (min)	Standard, μmol	Standard, area	Product, area	Product, μmol	Product %
0	263.69	1.00	0.00	0.00	0.00
60	263.69	5104.81	52.49	3.07	0.91
120	263.69	5546.93	93.09	5.01	1.48
1440	263.69	6494.56	1110.45	51.04	15.07
2870	263.69	4818.00	1530.64	94.83	28.00
4392	263.69	4818.48	2321.19	143.79	42.46
5891	263.69	5790.38	3168.65	163.34	48.23
7038	263.69	6623.67	3960.44	178.48	52.70

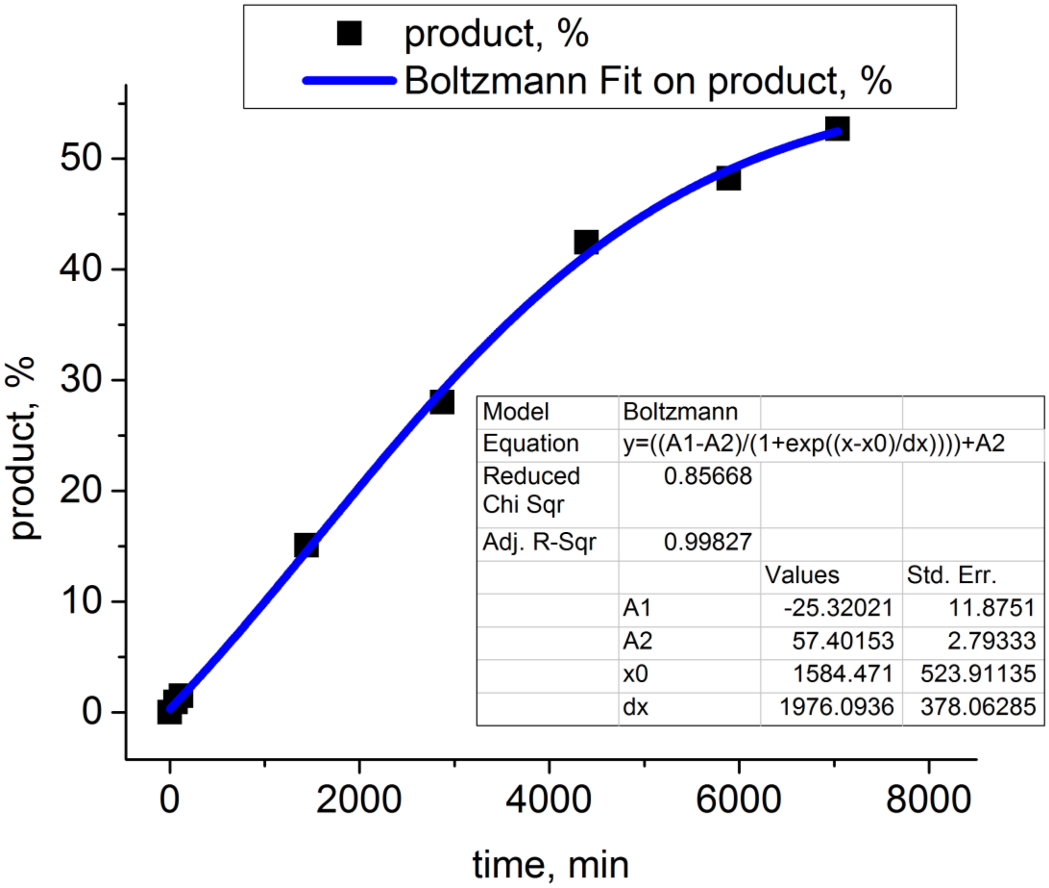

Interpolated $t_{1/2}$: 6169.13

Run 3

Time (min)	Standard, µmol	Standard, area	Product, area	Product, µmol	Product %
0	263.69	1.00	0.00	0.00	0.00
60	263.69	3850.09	94.13	7.30	2.16
195	263.69	3327.26	82.98	7.44	2.20
420	263.69	4594.59	209.15	13.59	4.02
1680	263.69	4864.01	869.43	53.35	15.79
3120	263.69	4522.60	1271.14	83.90	24.83
4560	263.69	7421.19	3004.45	120.84	35.77
6032	263.69	6193.00	3126.50	150.69	44.60
7456	263.69	6446.59	3657.44	169.35	50.12

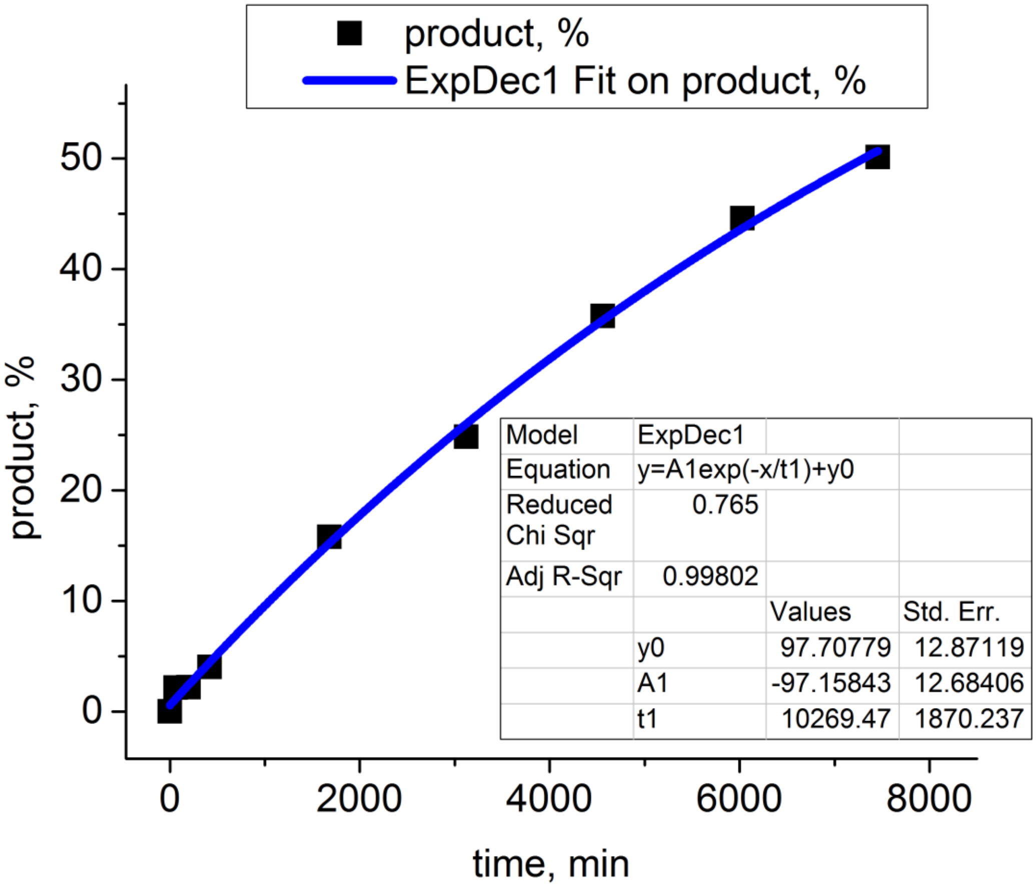

Interpolated $t_{1/2}$: 7330.06

Average $t_{1/2}$: 6302.73 min
Err $t_{1/2}$: 12.53%
Err $\log(t_{1/2})$: 0.017

Background

Run 1

Time (min)	Standard, μmol	Standard, area	Product, area	Product, μmol	Product %
**0**	263.69	1.00	0.00	0.00	0.00
**1449**	263.69	5766.35	712.91	36.90	10.93
**2880**	263.69	5195.09	1396.06	80.21	23.75
**4484**	263.69	3449.40	1072.49	92.81	27.48
**5760**	263.69	6488.74	2592.68	119.27	35.32
**7292**	263.69	6686.89	3015.04	134.59	39.86

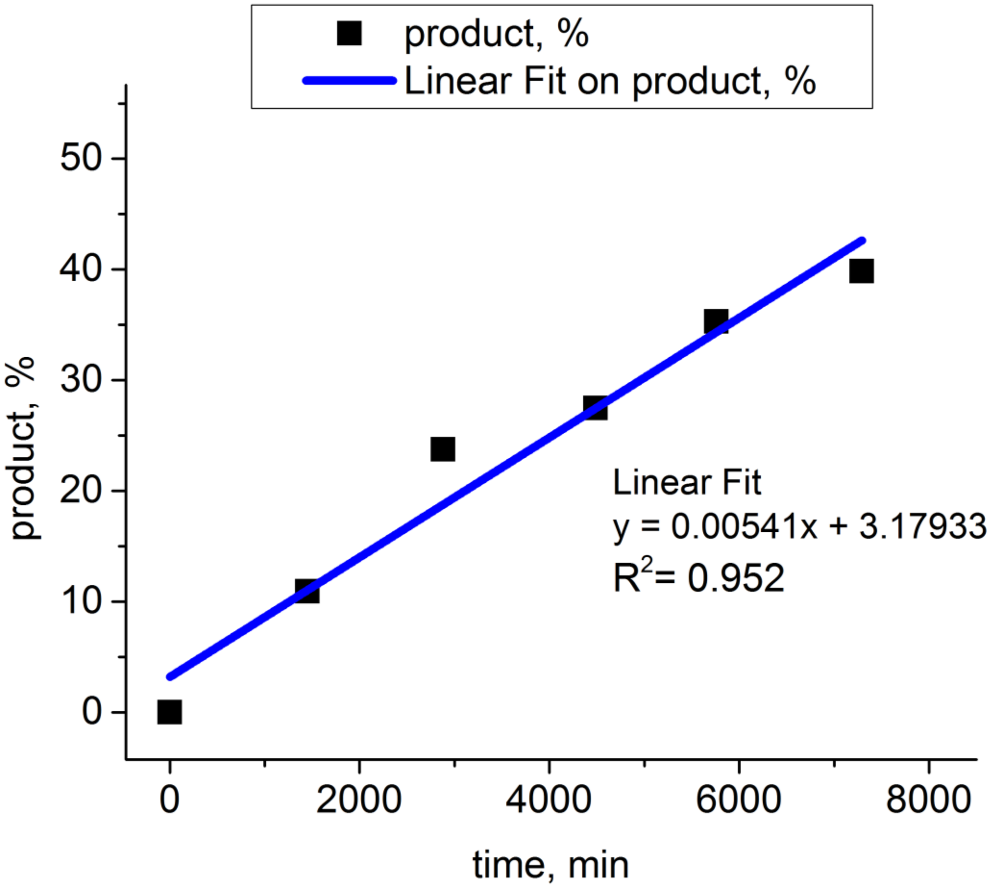

Interpolated $t_{1/2}$: 8670.50

Run 2

Time (min)	Standard, µmol	Standard, area	Product, area	Product, µmol	Product %
**0**	263.69	1.00	0.00	0.00	0.00
**1449**	263.69	6053.31	728.09	35.90	10.57
**2880**	263.69	5586.01	1248.33	66.71	19.64
**4484**	263.69	5452.73	1797.13	98.38	28.96
**5760**	263.69	5210.24	2155.05	123.46	36.35
**7292**	263.69	5565.54	2588.45	138.82	40.87

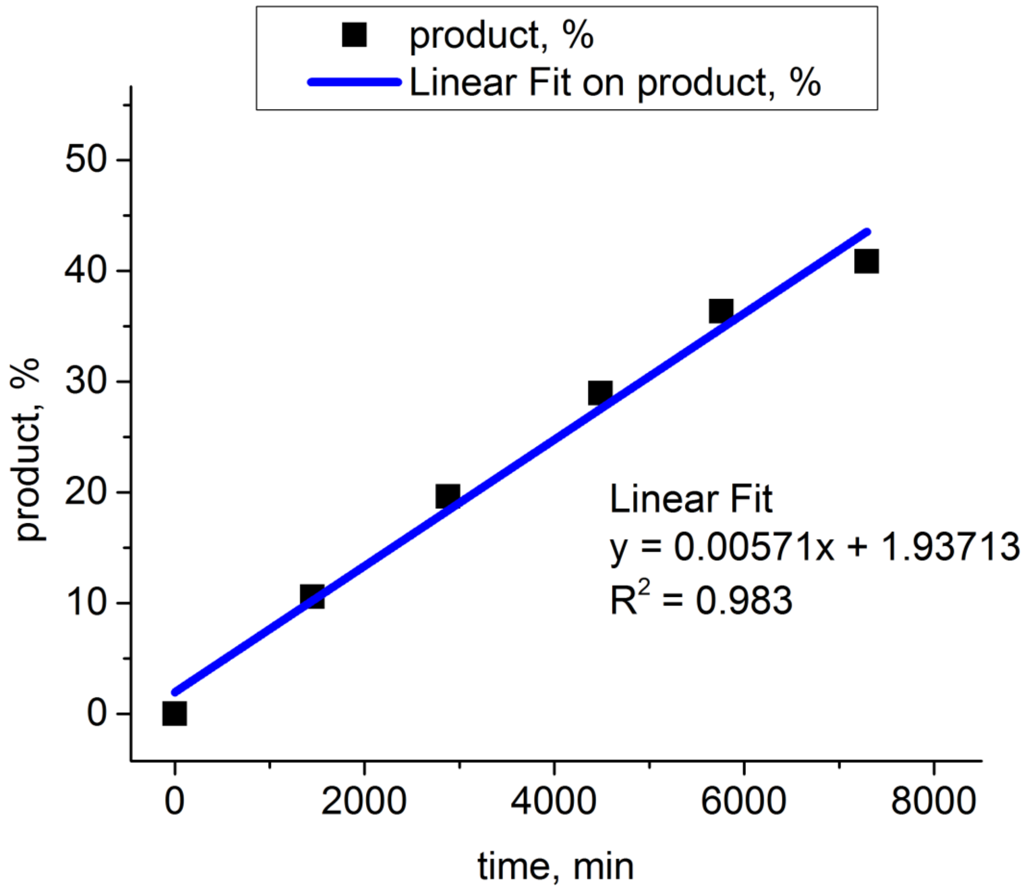

Interpolated $t_{1/2}$: 8432.09

Run 3

Time (min)	Standard, μmol	Standard, area	Product, area	Product, μmol	Product %
**0**	263.69	1.00	0.00	0.00	0.00
**2631**	263.69	5973.56	887.30	44.34	13.09
**3042**	263.69	4777.20	799.11	49.93	14.75
**4396**	263.69	7983.70	1954.97	73.09	21.59
**5871**	263.69	4706.25	1449.42	91.93	27.15
**7200**	263.69	5269.43	1879.08	106.44	31.43

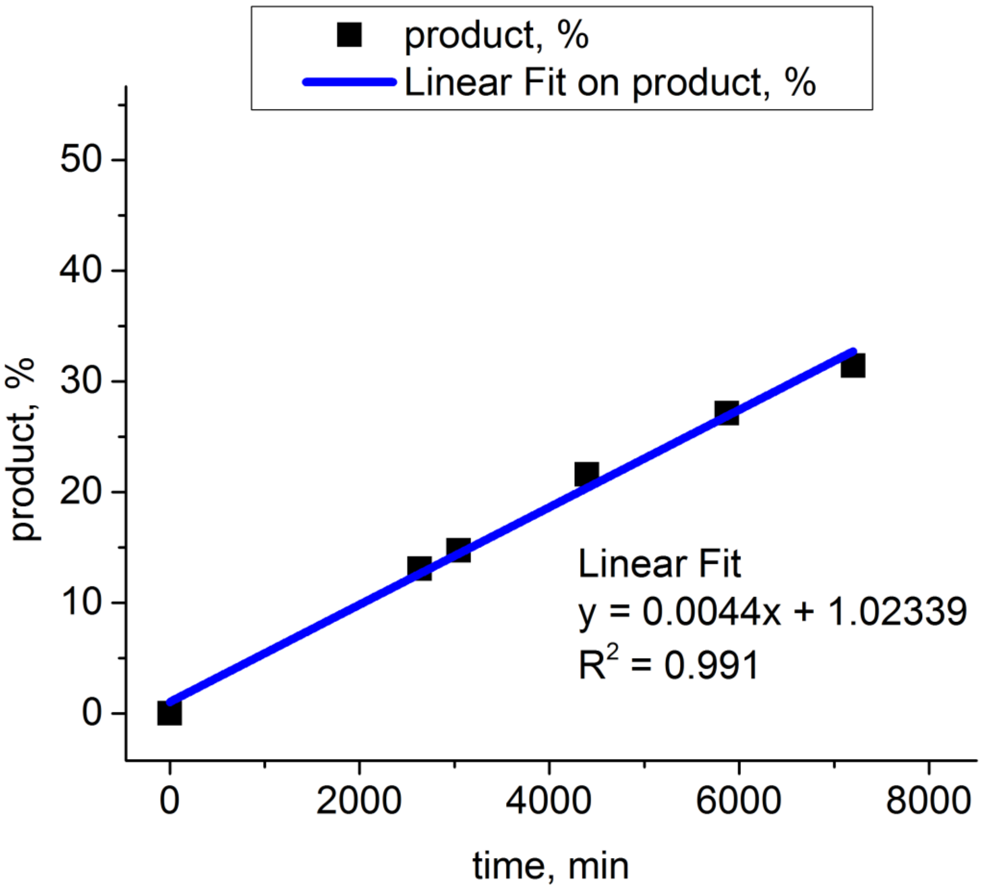

Interpolated $t_{1/2}$: 11131.05

Average $t_{1/2}$: 9411.21 min
Err $t_{1/2}$: 12.96%
Err $\log(t_{1/2})$: 0.0167

Computational Methods and Data
Descriptors

Most of the descriptors utilized in this study are included in the commercial Molecular Operating Environment package. Descriptors not included in the MOE package are available from the Chemical Computing Group web page via the SVL (scientific vector language) exchange with the exception of the quaternary ammonium surface area descriptor.[2] The main descriptor not available in the MOE package is the cross sectional area (XSA) descriptor.[3] Copyright prohibits the distribution of the SVL code in its entirety. That said, the code is simple and can be reassembled. But first, the SVL disclaimer to recognize that we did not invent this technology:

// COPYRIGHT (C) 2007-2009 CHEMICAL COMPUTING GROUP INC. ALL RIGHTS RESERVED.
//
// PERMISSION TO USE, COPY, MODIFY AND DISTRIBUTE THIS SOFTWARE IS HEREBY
// GRANTED PROVIDED THAT: (1) UNMODIFIED OR FUNCTIONALLY EQUIVALENT CODE
// DERIVED FROM THIS SOFTWARE MUST CONTAIN THIS NOTICE; (2) ALL CODE DERIVED
// FROM THIS SOFTWARE MUST ACKNOWLEDGE THE AUTHOR(S) AND INSTITUTION(S); (3)
// THE NAMES OF THE AUTHOR(S) AND INSTITUTION(S) NOT BE USED IN ADVERTISING
// OR PUBLICITY PERTAINING TO THE DISTRIBUTION OF THE SOFTWARE WITHOUT
// SPECIFIC, WRITTEN PRIOR PERMISSION; (4) ALL CODE DERIVED FROM THIS SOFTWARE
// BE EXECUTED WITH THE MOLECULAR OPERATING ENVIRONMENT (MOE) LICENSED FROM
// CHEMICAL COMPUTING GROUP INC.
//
// CHEMICAL COMPUTING GROUP INC. DISCLAIMS ALL WARRANTIES WITH REGARD TO THIS
// SOFTWARE, INCLUDING ALL IMPLIED WARRANTIES OF MERCHANTABILITY AND FITNESS,
// AND IN NO EVENT SHALL CHEMICAL COMPUTING GROUP INC. BE LIABLE FOR ANY
// SPECIAL, INDIRECT OR CONSEQUENTIAL DAMAGES OR ANY DAMAGES WHATSOEVER
// RESULTING FROM LOSS OF USE, DATA OR PROFITS, WHETHER IN AN ACTION OF
// CONTRACT, NEGLIGENCE OR OTHER TORTIOUS ACTION, ARISING OUT OF OR IN
// CONNECTION WITH THE USE OR PERFORMANCE OF THIS SOFTWARE.

Conformer Generation

For each tetraalkylammonium (R_4N^+) bromide salt, conformations within specified energy windows were generated using MOE 2013.08.[4] These conformational searches were produced using LowModeMD stochastic searches using MOE 2013.08 conformational search parameters. TEAB conformer generation was done using the **Systematic** method of conformer generation due to failure of the LowModeMD method to find multiple conformers.

General Procedure for Conformer Generation

The parameterization was set using MMFF94x in conjunction with the Born Solvation model using parameters for water solvation at 0 ºC (Inner dielectric: 1, outer dielectric constant: 88) (Figure 1). The rejection limit was set to 1000, RMSD limit set to 0.05, including hydrogens, and the energy window was set to 20 kcal/mol (Figure 2). Conformer searches were performed until convergence was determined when the average descriptor values arrived at a standard error <5%. Average XSA and NC4_SA was used instead of a Boltzmann weighted average based on calculated energies due to large number of conformers existing in easily reached energy ranges, as well as the unsolved problem of determining the optimal conformation of a molecular system with enormous degrees of freedom (such as THexDAB). Typical calculations time varied, between 0.5 h and 16 h depending on size of the compound and number of rotatable bonds.

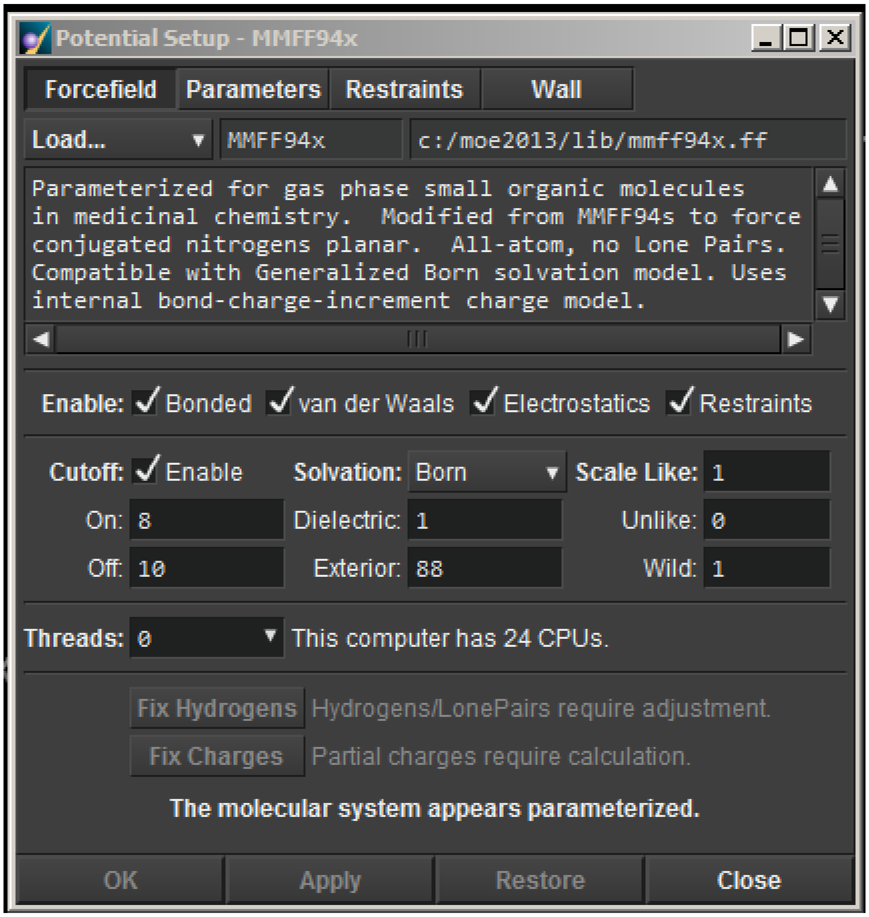

Figure 1. Parameterization in MOE 2013.08 for conformer searching.

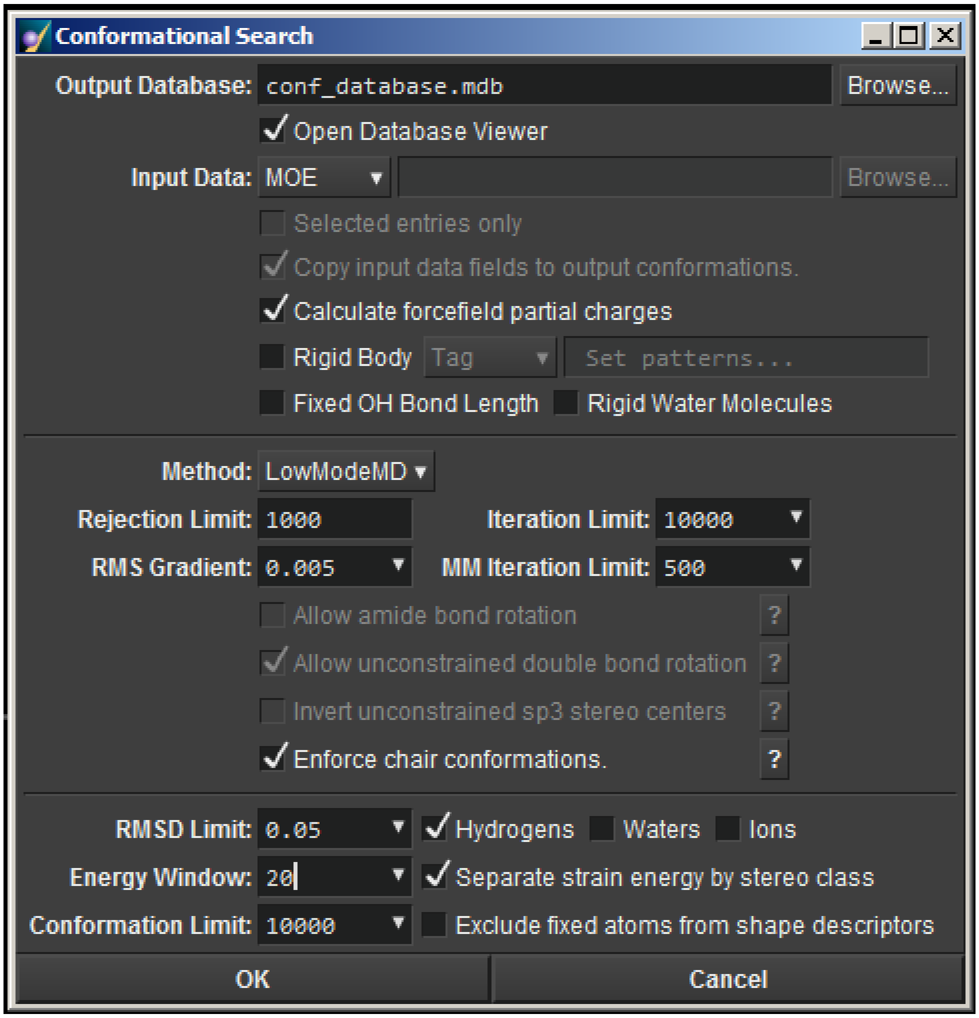

Figure 2. General settings for a conformational search using MOE2013.08.

XSA Descriptor Calculation

XSA calculations were performed using SVL code available on the SVL exchange.[3b] A typical set up is shown in Figure 3. The average XSA descriptor was calculated from the XSA values calculated for all conformations located in a conformational search within a 20 kcal/mol window.

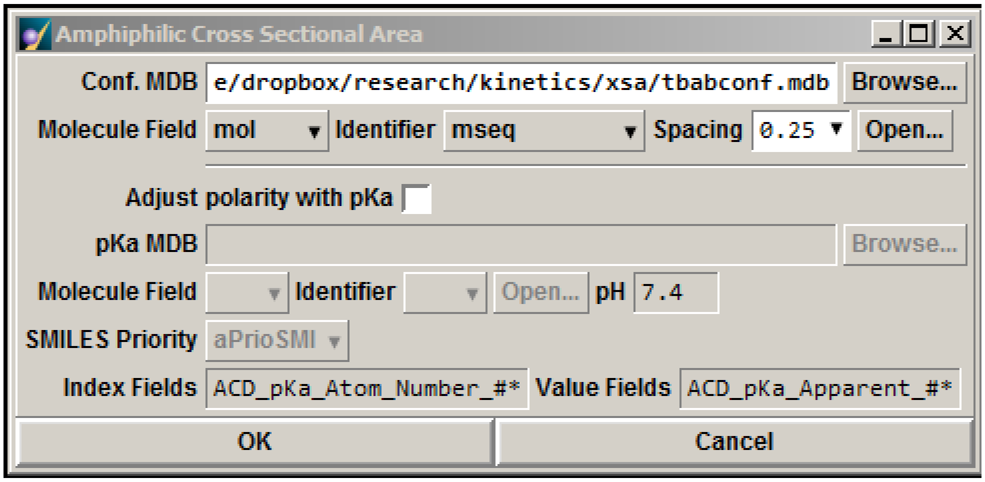

Figure 3. General settings for the XSA calculation.

Ammonium SA Descriptor Calculation with MOE

The solvent accessible ammonium surface area descriptor was calculated utilizing the following process.

Calculation Procedure:

First: searching for an ammonium ion with: n_cccc = sm_MatchAll ['[N+](C)(C)(C)C', all_atoms, []];

Second: separating heavy atoms from hydrogen using the code available in mol_surface_area.svl on the SVL exchange (by cw and db)

Third: selecting the ammonium ions with aSetSelected [n_cccc, 1]

Fourth: and calculating the surface area with: Descriptor = add AtomSurfaceArea[];

Table 1. Descriptor and Rate Data for R_4N^+ Salts.

Catalyst	XSA[a] (Å^2)	Ammonium Ion Accessibility (q)	NC4_SA[a] (Å^2)	log(k_{rel})[b]	Std. Err.[c]
**TMAB**	29.00	4.00	247.24	0.66	1.67%
**TEAB**	41.81	2.00	94.88	1.82	1.56%
**TBAB**	72.22	1.00	52.23	2.83	3.00%
**THAB**	101.17	0.67	48.22	3.02	4.50%
**THepAB**	114.14	0.57	48.26	3.00	7.53%
**TOAB**	128.83	0.50	47.68	2.89	5.75%
**TDAB**	152.03	0.40	44.64	2.06	2.27%
**TDoDAB**	174.34	0.33	39.10	1.36	0.67%
**THexDAB**	205.27	0.25	23.08	0.17	1.74%
**K^+ (background)**	17.31	NA	NA	0.00	1.67%

[a]Calculated using MOE2013.08 using MMFF94x as the calculation method and publically available SVL code plugins. [b]The value of log(k_{rel}) is obtained reporting the values of –log($t_{1/2}$) relative to –log($t_{1/2}$)$_{background}$. [c]Standard error is Stddev(avg(log(k_{rel}))/avg(log(k_{rel}))*100

Example XSA Values for Selected Chiral R_4N^+ Species

Catalyst	Number of Conformers	Avg XSA[a] (Å^2)	Std. Dev. (Å^2)	Min. XSA[a] (Å^2)	Max. XSA[a] (Å^2)
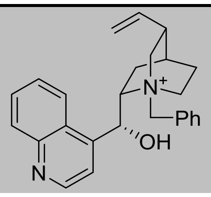	119	89.39	7.61	73.38	112.00
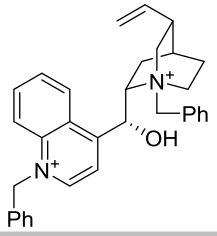	564	107.36	8.75	85.81	130.19
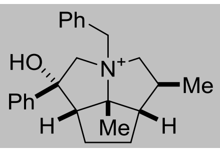	9	79.77	6.09	66.44	99.81
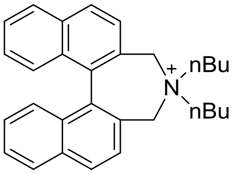	462	92.85	13.31	66.19	127.63
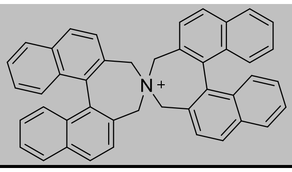	5	117.36	12.84	97.5	131.25

[a]Calculated using MOE2013.08 using MMFF94x as the calculation method and publically available SVL code plugins.

Tabulated Conformational Data for Descriptor Calculation

TEAB Conformational Descriptor Run Data

Run	Number of Conformers	Run Avg XSA[a] (Å^2)	Std. Dev. (Å^2)	Min. XSA[a] (Å^2)	Max. XSA[a] (Å^2)	Avg NC4_SA[a] (Å^2)	Std. Dev. NC4_SA (Å^2)
**1**	10	42.34	1.71	39.81	45.49	94.20	6.37
**2**	11	41.76	1.72	39.75	44.81	94.68	5.45
**3**	9	41.32	1.68	39.56	44.86	95.76	4.72
**Avg XSA**	41.81	**Std. Err.**[b]	1.00%	**Avg NC4_SA**	94.68	**Std. Err.**[c]	0.69%

[a]Calculated using MOE2013.08 using MMFF94x as the calculation method and publically available SVL code plugins. [b]Standard error is Stddev(XSA)/avg(XSA))*100. [c]Standard error is Stddev(NC4_SA)/avg(NC4_SA))*100.

TBAB Conformational Descriptor Run Data

Run	Number of Conformers	Run Avg XSA[a] (Å^2)	Std. Dev. (Å^2)	Min. XSA[a] (Å^2)	Max. XSA[a] (Å^2)	Avg NC4_SA[a] (Å^2)	Std. Dev. NC4_SA (Å^2)
**1**	379	72.52	6.19	53.38	88.13	52.44	7.46
**2**	370	72.63	6.72	55.06	88.19	51.75	7.22
**3**	221	71.52	6.06	55.50	86.19	52.49	6.67
**Avg XSA**	72.22	**Std. Err.[b]**	0.85%	**Avg NC4_SA**	52.23	**Std. Err.[c]**	0.65%

[a]Calculated using MOE2013.08 using MMFF94x as the calculation method and publically available SVL code plugins. [b]Standard error is Stddev(XSA)/avg(XSA))*100. [c]Standard error is Stddev(NC4_SA)/avg(NC4_SA))*100.

THAB Conformational Descriptor Run Data

Run	Number of Conformers	Run Avg XSA[a] (Å^2)	Std. Dev. (Å^2)	Min. XSA[a] (Å^2)	Max. XSA[a] (Å^2)	Avg NC4_SA[a] (Å^2)	Std. Dev. NC4_SA (Å^2)
**1**	4209	101.63	11.77	66.88	131.13	48.46	9.22
**2**	4244	101.35	11.73	63.94	129.94	48.06	9.29
**3**	4031	100.53	11.25	65.88	130.88	48.15	9.15
**Avg XSA**	101.17	**Std. Err.[b]**	0.46%	**Avg NC4_SA**	48.22	**Std. Err.[c]**	0.35%

[a]Calculated using MOE2013.08 using MMFF94x as the calculation method and publically available SVL code plugins. [b]Standard error is Stddev(XSA)/avg(XSA))*100. [c]Standard error is Stddev(NC4_SA)/avg(NC4_SA))*100.

THeptAB Conformational Descriptor Run Data

Run	Number of Conformers	Run Avg XSA[a] (Å^2)	Std. Dev. (Å^2)	Min. XSA[a] (Å^2)	Max. XSA[a] (Å^2)	Avg NC4_SA[a] (Å^2)	Std. Dev. NC4_SA (Å^2)
**1**	4926	113.97	13.79	69.56	151.69	48.24	9.25
**2**	4800	113.69	14.12	69.25	151.19	48.16	9.40
**3**	4825	114.77	13.88	72.00	154.31	48.39	9.13
**Avg XSA**	114.14	**Std. Err.[b]**	0.40%	**Avg NC4_SA**	48.26	**Std. Err.[c]**	0.20%

[a]Calculated using MOE2013.08 using MMFF94x as the calculation method and publically available SVL code plugins. [b]Standard error is Stddev(XSA)/avg(XSA))*100. [c]Standard error is Stddev(NC4_SA)/avg(NC4_SA))*100.

TOAB Conformational Descriptor Run Data

Run	Number of Conformers	Run Avg XSA[a] (Å^2)	Std. Dev. (Å^2)	Min. XSA[a] (Å^2)	Max. XSA[a] (Å^2)	Avg NC4_SA[a] (Å^2)	Std. Dev. NC4_SA (Å^2)
**1**	4971	129.25	16.90	72.44	171.69	48.09	9.57
**2**	5209	129.13	16.97	73.75	176.25	48.20	9.46
**3**	4974	128.11	15.92	80.25	173.00	46.74	10.09
**Avg XSA**	128.83	**Std. Err.[b]**	0.40%	**Avg NC4_SA**	47.68	**Std. Err.[c]**	1.38%

[a]Calculated using MOE2013.08 using MMFF94x as the calculation method and publically available SVL code plugins. [b]Standard error is Stddev(XSA)/avg(XSA))*100. [c]Standard error is Stddev(NC4_SA)/avg(NC4_SA))*100.

TDAB Conformational Descriptor Run Data

Run	Number of Conformers	Run Avg XSA[a] (Å^2)	Std. Dev. (Å^2)	Min. XSA[a] (Å^2)	Max. XSA[a] (Å^2)	Avg NC4_SA[a] (Å^2)	Std. Dev. NC4_SA (Å^2)
**1**	4938	150.81	20.56	86.38	206.69	43.32	10.79
**2**	5080	152.73	21.00	81.06	213.75	44.46	10.83
**3**	5088	152.54	21.02	87.94	209.69	46.15	10.73
**Avg XSA**	152.03	**Std. Err.[b]**	0.57%	**Avg NC4_SA**	44.64	**Std. Err.[c]**	2.60%

[a]Calculated using MOE2013.08 using MMFF94x as the calculation method and publically available SVL code plugins. [b]Standard error is Stddev(XSA)/avg(XSA))*100. [c]Standard error is Stddev(NC4_SA)/avg(NC4_SA))*100.

TDoDAB Conformational Descriptor Run Data

Run	Number of Conformers	Run Avg XSA[a] (Å^2)	Std. Dev. (Å^2)	Min. XSA[a] (Å^2)	Max. XSA[a] (Å^2)	Avg NC4_SA[a] (Å^2)	Std. Dev. NC4_SA (Å^2)
**1**	4451	175.39	22.91	91.25	245.94	39.83	11.74
**2**	4075	171.80	23.06	85.56	239.31	38.22	11.82
**3**	4108	175.82	23.27	101.5	249.44	39.25	11.94
**Avg XSA**	174.34	**Std. Err.[b]**	1.03%	**Avg NC4_SA**	39.10	**Std. Err.[c]**	1.70%

[a]Calculated using MOE2013.08 using MMFF94x as the calculation method and publically available SVL code plugins. [b]Standard error is Stddev(XSA)/avg(XSA))*100. [c]Standard error is Stddev(NC4_SA)/avg(NC4_SA))*100.

THexDAB Conformational Descriptor Run Data

Run	Number of Conformers	Run Avg XSA[a] (Å^2)	Std. Dev. (Å^2)	Min. XSA[a] (Å^2)	Max. XSA[a] (Å^2)	Avg NC4_SA[a] (Å^2)	Std. Dev. NC4_SA (Å^2)
**1**	3599	208.02	29.42	123.19	313.5	25.98	12.83
**2**	1933	203.85	27.21	133.13	314.25	21.70	14.22
**3**	2728	203.95	27.77	121.31	298.44	21.60	12.08
**Avg XSA**	205.27	**Std. Err.[b]**	0.95%	**Avg NC4_SA**	23.08	**Std. Err.[c]**	2.02%

[a]Calculated using MOE2013.08 using MMFF94x as the calculation method and publically available SVL code plugins. [b]Standard error is Stddev(XSA)/avg(XSA))*100. [c]Standard error is Stddev(NC4_SA)/avg(NC4_SA))*100.

Model Generation

Model generation was typically done using Origin 9 Pro fitting analysis algorithms. Parabolic, polynomial, reciprocal, and bilinear fitting can be achieved using the available software options in Origin Pro 9.[5]

For kinetic run $t_{1/2}$ interpolation, the ExpDec1 fitting model was used to find an exponential decay equation and allow interpolation of the estimated time 50% with respect to product conversion was observed.

XSA Cubic Model

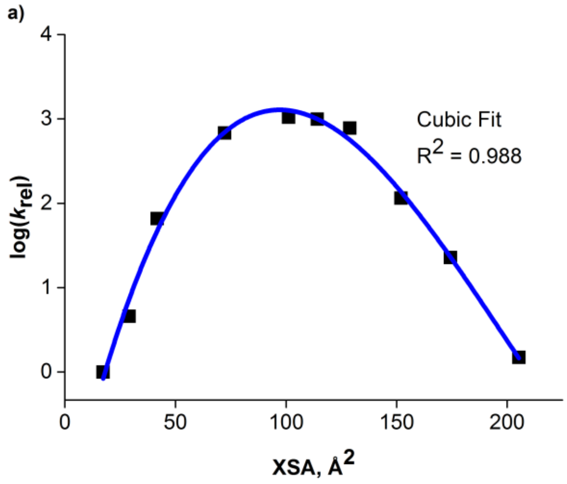

$$\log(k_{rel}) = 1.322 * 10^{-6}(XSA)^3 - 7.795 * 10^{-4}(XSA)^2 + 0.114(XSA) - -1.825 \quad \text{(Eq. 1)}$$

XSA Parabolic Model

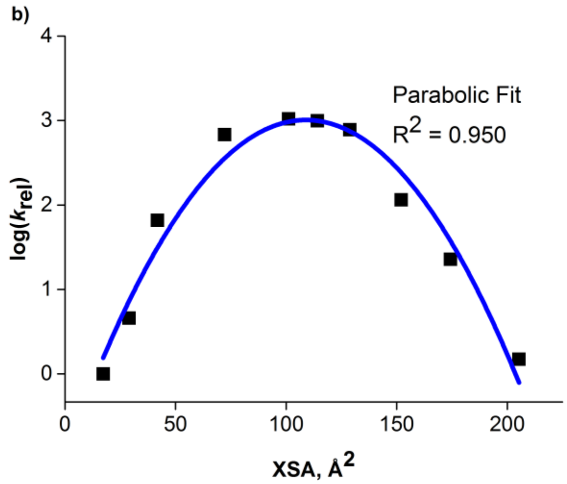

$$log(k_{rel}) = -3.351 * 10^{-4}(XSA)^2 + 0.0730(XSA) - 0.974 \quad \text{(Eq. 2)}$$

XSA Bilinear Model

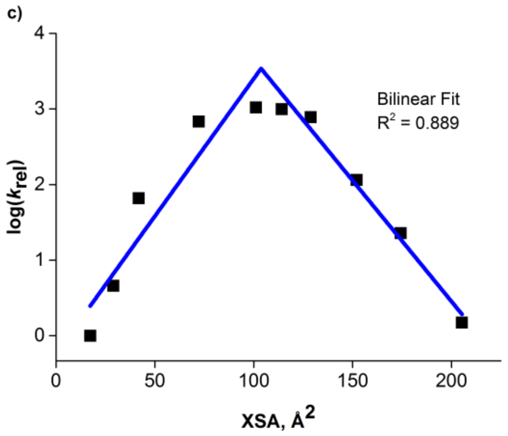

$$\log(k_{rel}) = \begin{cases} \frac{y_1(x_3 - XSA) + y_3(XSA - x_1)}{x_3 - x_1}, & if\ XSA < x_3 \\ \frac{y_3(x_2 - XSA) + y_2(XSA - x_3)}{x_2 - x_3}, & if\ XSA \geq x_3 \end{cases} \quad \text{(Eq. 3)}$$

**x_1**	17.31	**y_1**	0.394
**x_2**	205.27	**y_2**	0.285
**x_3**	103.70	**y_3**	3.535

XSA 4th Order Polynomial Model

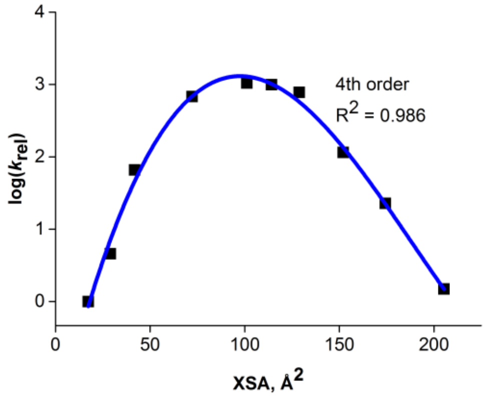

$$\boldsymbol{log(k_{rel}) = 1.351 * 10^{-9}(XSA)^4 + 7.174 * 10^{-7}(XSA)^3 - 6.901 * 10^{-4}(XSA)^2 + 0.1091(XSA) - 1.750} \quad \text{(Eq. 4)}$$

NC4_SA Bilinear Model

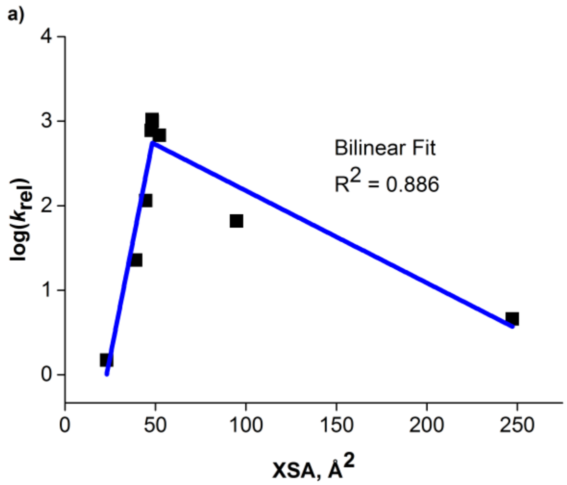

$$\log(k_{rel}) = \begin{cases} \frac{y_1(x_3 - NC4\_SA) + y_3(NC4\_SA - x_1)}{x_3 - x_1}, & if\ NC4\_SA < x_3 \\ \frac{y_3(x_2 - NC4\_SA) + y_2(NC4\_SA - x_3)}{x_2 - x_3}, & if\ NC4\_SA \geq x_3 \end{cases} \quad \text{(Eq. 5)}$$

$\mathbf{x_1}$	23.08	$\mathbf{y_1}$	0.00645
$\mathbf{x_2}$	247.24	$\mathbf{y_2}$	0.571
$\mathbf{x_3}$	48.25	$\mathbf{y_3}$	2.744

Bilinear Model with *q*

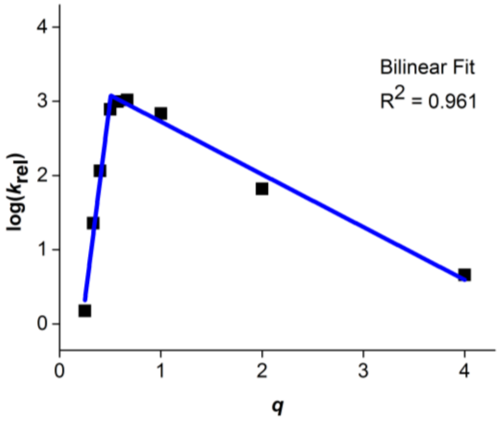

$$\log(k_{rel}) = \begin{cases} \frac{y_1(x_3 - \boldsymbol{q}) + y_3(\boldsymbol{q} - x_1)}{x_3 - x_1}, & if\ \boldsymbol{q} < x_3 \\ \frac{y_3(x_2 - \boldsymbol{q}) + y_2(\boldsymbol{q} - x_3)}{x_2 - x_3}, & if\ \boldsymbol{q} \geq x_3 \end{cases} \quad \text{(Eq. 6)}$$

$\mathbf{x_1}$	0.17249	$\mathbf{y_1}$	-0.51741
$\mathbf{x_2}$	3.95078	$\mathbf{y_2}$	0.62883
$\mathbf{x_3}$	0.5059	$\mathbf{y_3}$	3.07717

XSA Correlation with *q*

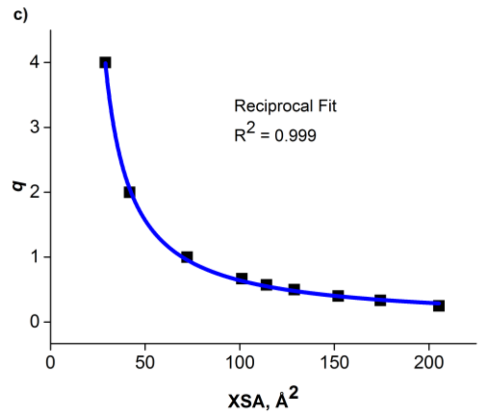

$$q = \frac{1}{0.01839(XSA) - 0.28261} \quad \text{(Eq. 7)}$$

XSA Correlation with Carbon Chain Length (Cn)

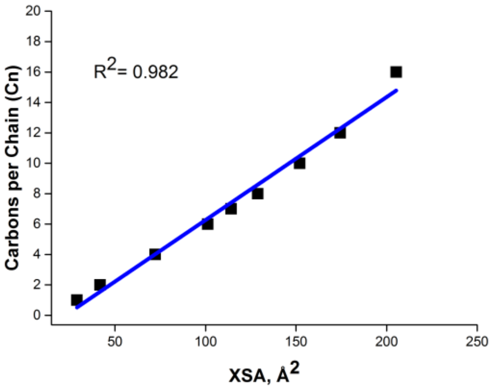

$$Cn = 0.00383(XSA) - 1.825 \quad \text{(Eq. 8)}$$

XSA Model Selection

In order to determine the best overall model between XSA and rate, the Akaike information criterion (AIC) and Bayesian information criterion (BIC) values were calculated for each model. These statistical values allow the comparison of models to determine the best compromise of model complexity and goodness of fit. The equations for each are given below. These values are computed using OriginPro 9's implementation[5] of AIC and BIC. The number of inputs is denoted *N* and the number of parameters in the model is denoted as *K*. BIC places a higher penalty on over-fitting models, and thus both AIC and BIC were considered. For both AIC and BIC, the minimum value of each is considered for the best model. By both AIC and BIC, the cubic model is predicted to be the best model of the set of models containing linear, bilinear, parabolic, cubic, and 4th order polynomic models.

$$AIC = \begin{cases} Nln\left(\frac{RSS}{N}\right) + 2K, & when \frac{N}{K} \geq 40 \\ Nln\left(\frac{RSS}{N}\right) + 2K + \frac{2K(K+1)}{N-K-1}, & when \frac{N}{K} < 40 \end{cases} \quad \text{(Eq. 9)}$$

$$BIC = Nln\left(\frac{RSS}{N}\right) + Kln(N) \quad \text{(Eq. 10)}$$

AIC and BIC Comparison of XSA and Rate Models[a]

Model	N	K	RSS	AIC	BIC
Linear	**10**	**2**	**12.52008**	**12.247**	**9.155**
Bilinear	**10**	**4**	**0.93505**	**1.303**	**-12.184**
Parabolic	**10**	**3**	**0.49079**	**-14.143**	**-20.933**
Cubic	**10**	**4**	**0.09751**	**-21.303**	**-34.790**
4th order polynomial	**10**	**5**	**0.09646**	**-6.412**	**-32.596**

[a]Calculated using OriginPro 9 model comparison AIC and BIC algorithms.

References

1 Standard error is StdDev($t_{1/2}$)/Average($t_{1/2}$) where $t_{1/2}$ is the set of $t_{1/2}$ values for a single catalyst, and reported as a percentage. Log standard error is StdDev(log($t_{1/2}$))/Average(log($t_{1/2}$)) where log($t_{1/2}$) is the set of log($t_{1/2}$) values for a single catalyst.

2 http://svl.chemcomp.com/gl/index.php

3 (a) S.A. Wildman and G.M. Crippen, *J. Chem. Inf. Comput. Sci.* 1999, **39**, 868-873. (b) The descriptor can be found on the SVL exchange at http://svl.chemcomp.com/filedetails.php?lid=651&cid=43.

4 *Molecular Operating Environment (MOE)*, 2013.08; Chemical Computing Group Inc., 1010 Sherbooke St. West, Suite #910, Montreal, QC, Canada, H3A 2R7, 2014.

5 Origin (OriginLab, Northampton, MA)
